# Promoting independence in Lewy body dementia through exercise: the PRIDE study

**DOI:** 10.1186/s12877-022-03347-2

**Published:** 2022-08-09

**Authors:** Michael J. Inskip, Yorgi Mavros, Perminder S. Sachdev, Jeffrey M. Hausdorff, Inbar Hillel, Maria A. Fiatarone Singh

**Affiliations:** 1 Sport and Exercise Science, College of Healthcare Sciences, James Cook University, Townsville, QLD Australia; 2 Exercise and Sport Science, School of Health Sciences, Faculty of Medicine and Health, The University of Sydney, Camperdown, NSW 2006 Australia; 3 Centre for Healthy Brain Ageing (CHeBA), Discipline of Psychiatry, University of New South Wales, Sydney, NSW Australia; 4 Neuropsychiatric Institute, The Prince of Wales Hospital, Sydney, NSW Australia; 5 Center for the Study of Movement, Cognition and Mobility, Neurological Institute, Tel Aviv Sourasky Medical Center, Tel Aviv, Israel; 6 Sagol School of Neuroscience and Department of Physical Therapy, Sackler Faculty of Medicine, Tel Aviv University, Tel Aviv, Israel; 7 Rush Alzheimer’s Disease Center and Department of Orthopaedic Surgery, Rush University Medical Center, Chicago, Illinois USA; 8 Sydney Medical School, The University of Sydney, Camperdown, NSW Australia; 9 Hebrew SeniorLife, Boston, MA USA

**Keywords:** Lewy body, Anabolic exercise, Frailty, Functional independence, Exercise physiology

## Abstract

**Background:**

Lewy body dementia (LBD) is an aggressive type of dementia of rapid, fluctuating disease trajectory, higher incidence of adverse events, and poorer functional independence than observed in Alzheimer’s disease dementia. Non-pharmacological treatments such as progressive, high-intensity exercise are effective in other neurological cohorts but have been scarcely evaluated in LBD.

**Methods:**

The Promoting Independence in Lewy Body Dementia through Exercise (PRIDE) trial was a non-randomised, non-blinded, crossover pilot trial involving older adults with LBD consisting of a baseline assessment, an 8-week wait-list, and an 8-week exercise intervention. The aims of this study were to evaluate the determinants of the primary outcome functional independence, as measured by the Movement Disorder Society Unified Parkinson’s Disease Rating Scale, and the feasibility and preliminary efficacy of an exercise intervention on this outcome. Additionally, important clinical characteristics were evaluated to explore associations and treatment targets. The exercise intervention was supervised, clinic-based, high-intensity progressive resistance training (PRT), challenging balance, and functional exercises, 3 days/week.

**Results:**

Nine participants completed the baseline cross-sectional study, of which five had a diagnosis of Parkinson’s disease dementia (PDD), and four dementia with Lewy Bodies (DLB). Six completed the exercise intervention (three PDD, three DLB). The cohort was diverse, ranging from mild to severe dementia and living in various residential settings. Greater functional independence at baseline was significantly associated with better physical function, balance, cognition, quality of life, muscle mass ratio, walking endurance, faster walking speed and cadence, and lower dementia severity (*p* < 0.05). Participants declined by clinically meaningful amounts in functional independence, cognition, physical function, muscle mass, and weight over the wait-list period (*p* < 0.05). Following exercise, participants improved by clinically meaningful amounts in functional independence, cognition, physical function, and strength (*p* < 0.05). Progressive, high intensity exercise was well-tolerated (> 80% adherence), and only one minor exercise-related adverse event occurred.

**Conclusions:**

PRIDE is the first exercise trial conducted specifically within individuals diagnosed with LBD, and provides important insight for the design of larger, randomized trials for further evaluation of progressive, high-intensity exercise as a valuable treatment in LBD.

**Trial registration:**

The PRIDE trial protocol has previously been prospectively registered (08/04/2016, ANZCTR: ACTRN12616000466448).

**Supplementary Information:**

The online version contains supplementary material available at 10.1186/s12877-022-03347-2.

## Background

Lewy body dementia (LBD) is an aggressive neurodegenerative disorder involving cognitive impairment, psychosis, parkinsonism and autonomic disturbances that cause a progressive decline in functional independence [1]. The term includes either of two diagnoses; dementia with Lewy bodies (DLB) whereby the dementia occurs at the same time or within one year of onset of parkinsonism, and Parkinson’s disease dementia (PDD) whereby the dementia occurs more than one year following a diagnosis of Parkinson’s disease (PD) [1]. Lewy body dementia is the second most prevalent type of dementia, accounting for between 15 to 24% of all people diagnosed with dementia in clinical settings [2, 3]. Compared with Alzheimer’s disease dementia (AD, 60–70% of all diagnoses) [4], older adults with LBD experience faster cognitive decline [5], lower physical activity levels [6], higher risk of falls [7], delirium [8], malnutrition [9], and frailty [10], earlier residential aged care admission, higher care costs [5], and an average survival of 1.6 years less after diagnosis [11]. Additionally, individuals with LBD have poorer functional independence in daily living, which is associated with lower quality of life and faster disease trajectory [5, 6].

Current treatments for LBD are symptomatic and focused predominantly on pharmaceuticals [1], with scarce evaluation of non-pharmacological treatments such as exercise [1, 12, 13]. Medications such as donepezil and rivastigmine are effective for cognitive impairment in mild disease, however they often become less effective later in the disease course [14]. Furthermore, neuroleptics prescribed for psychosis increase the risk of falls, serious adverse events and premature mortality in LBD [14]. Additionally, those with LBD are more likely to experience polypharmacy (≥ 5 prescribed medications), which increases the risk of frailty and functional decline, for which there is no current pharmaceutical treatment [5, 8].

Conversely, non-pharmacological treatments such as progressive, high-intensity exercise offer a viable, effective treatment for frailty in older adults [15] and could benefit those with LBD. In similar cohorts with PD and AD, progressive, high-intensity exercise improves physical function, strength, cognition, affect, and functional independence safely [16, 17]. However no randomized controlled trials (RCTs) of exercise have been published in LBD [12] and the majority of exercise trials within dementia or PD exclude individuals with LBD, since they have both cognitive *and* motor impairments [12]. Thus, there is a need, recognised by recent LBD guidelines [1], for trials to evaluate the effects of exercise specifically within older adults living with LBD.

The *Promoting Independence in Lewy Body Dementia through Exercise* (PRIDE) trial [18] is the first study to specifically evaluate the effects of a progressive, high-intensity exercise program in LBD.

The aims of the study were to:

1. Identify determinants of functional independence in individuals living with LBD that may be amenable to a targeted exercise intervention
2. Assess the feasibility, including adoption and adherence, adverse events, and preliminary efficacy of this evidence-based exercise program in individuals with LBD.

The hypotheses were:

1. Low muscle strength and balance will be associated with functional dependency in individuals with LBD at baseline.
2. A progressive, high-intensity exercise intervention targeting strength and balance will improve functional independence in individuals with LBD.

## Methods

### Study design

The PRIDE trial protocol has previously been published [18] and prospectively registered (08/04/2016, ANZCTR: ACTRN12616000466448) [19]. PRIDE was a non-randomised, non-blinded, crossover pilot trial involving older adults with LBD consisting of a baseline assessment (carried out in participant’s home) then 8-week wait-list, and subsequently an 8-week exercise intervention in the clinic (Cumberland Campus, University of Sydney, Lidcombe, Australia). All participants undertook the 8-week wait-list period prior to crossing over to exercise intervention to avoid the need for a washout period that would have otherwise been required if some participants performed the exercise intervention first, due to the anticipated residual effects of exercise.

### Ethics

Ethical approval was obtained from the University of Sydney Human Research Ethics (HREC 2: 2016/209). Written informed consent was obtained for all caregivers and participants. For participants unable to provide informed consent due to cognitive impairment, caregivers consented on their behalf. PRIDE adhered to the CONSORT guidelines for pilot trials [20].

#### Participant recruitment

Recruitment began in April 2016. Study information was disseminated to local geriatricians, neurologists, General Practioners (GPs), dementia and PD support groups and networks in the Sydney metropolitan area, and participants or their caregivers contacted the study investigators if interested in taking part. All of the participants discovered the study through dementia or PD support groups.” The inclusion and exclusion criteria have been previously described [18].

#### Screening procedure

Participants and/or their caregivers were screened over the telephone via a 1-h screening questionnaire to determine eligibility for the PRIDE trial and were read the Participant Information Statement. Questions relating to demographics (inclusive of caregiver), study eligibility, physical activity, current health status, prior and current injury and illness, prescribed medications, and medical professionals associated with care of the participant were asked of the caregiver/participant dyad. Medical information was sought from participants’ GPs or specialists after obtaining consent to further clarify eligibility as required. Additionally, comprehensive geriatric assessment of each participant was performed by the study geriatrician (M.F.S.) prior to commencing baseline one-repetition maximum (1RM) strength testing and exercise intervention. This assessment included taking a thorough medical and social history, current medications, review of systems, physical examination and a request to the GP if further information or testing was required.

#### Estimated sample size

Based upon similar cross-sectional studies in PD, to show moderate correlations (*r* = 0.5) with β = 0.20 (power of 0.8) and α = 0.05 for the baseline cross-sectional analysis, we calculated a minimum of 30 participants would be needed, inclusive of a 20% expected attrition rate [18].

#### Assessment procedures

The study coordinator (M.I.), an accredited exercise physiologist (AEP), performed all assessments and interventions except for the physician screen performed by the study geriatrician.

### Intervention

#### Wait-list period

Participants and caregivers continued normal daily activities and participants were monitored weekly for adverse events, status, and medication changes.

#### Exercise intervention

High intensity, progressive exercise training was conducted in the medically-supervised university clinic one-on-one by an AEP, 3 days/week for 60-min sessions.

Training sessions were divided into four sections: *static balance, dynamic balance, functional practice, and progressive resistive exercis*e performed in that order to minimise fatigue in the participants. Comprehensive details of this training program are described in the protocol [18] and Supplementary Text S1, Additional File 1.

#### Adverse events

Adverse events, health status and medical care/interventions were monitored via weekly telephone caregiver questionnaires and additional information was gathered from participants’ doctors, if required. Adverse events were defined a priori and included any exacerbation of underlying disease, or new onset musculoskeletal, cardiovascular or metabolic abnormalities. The study geriatrician and ethics committee evaluated all adverse events to adjudicate all events as potentially/definitely related to the study exercise or assessment protocols or not, or any need to change the study protocol.

#### Following trial completion

Participants were invited to continue supervised exercise within the clinic with no additional cost or time limit after the trial completion.

### Measures

The assessment battery was selected to evaluate the contributions of a wide range of factors potentially related to functional independence in LBD [18].

#### Primary outcome

Functional independence was measured via the total score of the Movement Disorder Society Unified Parkinson’s Disease Rating Scale (MDS-UPDRS) [21], an effective tool for evaluating disease severity, disability and independence in parkinsonian disorders including LBD [22]. Minimum clinically important difference (MCID) on this scale is 4.7 points [23].

#### Secondary outcomes

Measures including *cognition, psychosocial function, quality of life, cardiovascular status, body composition, health status*, *medication interactions*, *physical performance, exercise capacity and additional functional independence* measures were assessed along with *caregiver outcomes including burden and psychosocial state* (see protocol) [18].

Additionally, *physical activity and sedentary behaviour* variables were derived from a small, lumbar-mounted accelerometer *(*Axivity AX3, York, UK; dimensions 23.0 × 32.5 × 7.6 mm; weight: 11 g; accuracy 20 parts per million) by co-investigators (*J.H., I.H*). Supplementary Text S1, Additional File 1 provides a thorough description of these variables.

### Statistical analysis

Full statistical methods are detailed in Supplementary Text S1, Additional File 1. Data analysis was performed using data analysis software (IBM Corp. Released 2017. IBM SPSS Statistics for Windows, Version 26.0. Armonk, NY). Statistical significance was defined as α < 0.05 for all analyses. Visual box plot inspection and the Shapiro-Wilke test determined that the data were not normally distributed and therefore non-parametric statistics were conducted. Descriptive data are presented as median (range) or frequencies as appropriate, and Spearman’s correlation used to evaluate baseline associations. Strength of the association was interpreted as small ≤  ± 0.2—< 0.5, moderate = 0.5- < 0.8, and strong ≥  ± 0.8. Additionally, the Wilcoxon signed rank test was used to separately analyse changes scores for [1] baseline to pre-intervention, and 2) pre-intervention to post-intervention, to utilise all available data. Hodge-Lehmann’s estimators provided a median change and confidence interval set at 95% upper and lower bounds. For each individual, accelerometry-derived values reflect median values of the daily mean for the week of monitor wear as the data were not normally distributed at the individual level across the 7 days.

## Results

### Recruitment and retention

Thirteen participant/caregiver dyads contacted the study team (May 2016 to December 2017). Nine participants were eligible for baseline testing, and subsequently enrolled into the 8-week wait-list period. Six participants subsequently completed the 8-week exercise intervention, with two dropping out due to ill health unrelated to the intervention (Supplementary Text S1). An additional participant completed the intervention period after a 9-month delay due to multiple clinical events unrelated to the study. His intervention results are reported separately in a published case report [24].

### Adverse events

Three adverse events were reported during the wait-list period for three separate participants and were adjudicated unrelated to the study. Two adverse events were reported during the exercise intervention. The first was delirium secondary to faecal impaction unrelated to the study but which led to participant’s withdrawal from the intervention. The second, a temporary exacerbation of a pre-existing inguinal hernia, was adjudicated likely related to the study, however, the participant completed the intervention following slight modification of the exercises (Supplementary Text S1, Additional File 1).

### Baseline characteristics

The characteristics of the cohort at baseline are presented in Table 1 and a detailed description provided in Supplementary Text S1, Additional File 1. Seven out of nine participants were male, eight were white non-Hispanic, and one Hispanic. All but two participants were living with dementia for > 12 months, and five participants diagnosed with PDD had been living with PD for 4 -17 years prior to the dementia diagnosis. Seven participants were prescribed dopaminergic medications (median levodopa equivalent dose (LED) of 450.0 mg, range 26.0 – 1297.5 mg). A neurologist diagnosed all but two participants. Five participants resided at home, one in an independent aged care unit, and three in aged care facilities.

**Table 1:** Baseline characteristics of participants

			*n* = 9		
Age (years)			74	(66–84)	
Sex (male), *n*			7		
Ethnicity (Caucasian), *n*			8		
Body Mass Index (BMI), kgm^−2^			24.9	(21.0–26.3)	
Diagnosis (PDD/DLB), *n*			5 / 4		
Time since diagnosis^a^, *months*			12	(3–48)	
Visits required for diagnosis^b^, *n*			3	(1–7)	
MDS-UPDRS total score, */260*			86	(57–169)	
Part III motor—sub score, */132*			46	(33–82)	
Clinical dementia rating (CDR) score^c^, */3*					
Mild (1), *n*			5		
Moderate (2), *n*			2		
Severe (3), *n*			2		
FIM total score, */126*			102	(30–122)	
MMSE total score, */30*			22	(5–29)	
PD-CRS total score, */134*			44	(7–83)	
GDS–15 total score, */15*			1	(0–3*)	
Reported falls in prior year^d^, *n*			2	(0–20)	
Injurious falls requiring hospitalization ^e^, *n*			5		
Recurrent fallers (≥ 2 falls in last year) ^f^, *n*			6		
Diagnosed comorbidities ^g^, *n*			5		
Prescribed medications ^h^, *n*			5		
Participants with:					
≥ 5 medications prescribed (polypharmacy), *n*			6		
Anticholinergic Burden (ACB) score ≥ 3, *n*			2		
Potentially inappropriate medications (PIMs), *n*			5		
*Medication class (number of participants taking* ≥ *1 medication in each class)*					
Dopaminergic, *n*	7	Anti-platelet, Anti-coagulant, *n*			3
Neuroleptic, *n*	2	Statins, *n*			3
Sedative/Tranquilizer, *n*	2	Blood pressure regulating, *n*			4
Antidepressant, *n*	4	PPI, *n*			2
AChEI, /NMDA receptor agonist, *n*	5	Supplements, *n*			4

All values a presented as median (range) or as n participants satisfying criteria. Higher scores in the MDS-UPDRS, CDR, GDS-15 and ACB; and lower scores on the FIM, MMSE, and PD-CRS indicate worse performance on that measure respectively. *PDD* Parkinson’s disease dementia, *DLB* Dementia with Lewy bodies, *MDS-UPDRS* Movement Disorder Society Unified Parkinson’s Disease Rating Scale. (21) Total score includes parts I-IV, Part III is the assessor rated motor score, *FIM* Functional independence measure [25], *MMSE* Mini-mental state exam [26], *PD-CRS* Parkinson’s disease Cognitive rating scale [27], *GDS-15* Geriatric Depression Scale – 15 item [28], *AChEI/NMDA* Acetyl-cholinesterase Inhibitor/ N-methyl-D-aspartate, *PPI* Proton Pump Inhibitor. ^a^ denotes the months since the participants received a formal diagnosis of LBD. ^b^ denotes the number of healthcare visits required to reach a formal diagnosis of LBD. ^c^ The CDR algorithm score (0–3) is derived from a sum score (0–18) [29] ^d^ denotes the median number of reported falls per participants. ^e^ Accumulative total of falls resulting in injury and subsequent medical treatment across the cohort. ^f^ Number of participants who had two or more falls in previous 12 months. ^g^ previously diagnosed conditions and conditions identified within physician screen at baseline assessment. ^h^ Number of medications including prescribed supplements. Polypharmacy is defined as ≥ 5 prescribed medications. ACB is a scale assessing the combined Anticholinergic risk from various medications, whereby the score is the added total of all medications with possible (1 point), or definite (2–3 points) anticholinergic side effects [30]

Four participants had comparable functional independence to older adults with advanced PD (Hoehn & Yahr stage IV, mean Functional Independence Measure (FIM) total score 45.5 ± 13.7) [31] while five were more independent. Four participants had scores on the Mini-mental State Examination (MMSE) [26] that were above the suggested cut off for dementia of ≤ 24/30, [26]. However, all but one participant had cognitive scores consistent with dementia (scored ≤ 80/134) in the Parkinson’s Disease Cognitive Rating Scale (PD-CRS) [27]. Dementia severity was mild to moderate in all but two participants according to the Clinical Dementia Rating (CDR) algorithm score, [29] with the remaining two scoring in the severe range. There were 45 falls reported by caregivers in the 12 months prior to study contact among six participants, with only five of these falls warranting medical attention. Additionally, four participants met criteria for sarcopenia, [32] and all but one participant was either pre-frail or frail [15] (Supplementary Table S2, Additional File 1).

For accelerometry-derived values (Supplementary Table S3, S4, and Figure S2, Additional File 1), daily physical activity varied greatly, with participants spending 2.15 h (range 2 – 7.7 h) active and 11.4 h (range 9.2 – 13.7 h) physically inactive on average. Cadence varied considerably outside of the typical physiological range of 100–115 steps/min, with one participant below this range, and four above. Stride time variability was significantly higher (worse) than reported in PD cohorts, with seven participants having a median variability exceeding the mean value reported for individuals with PD characterised as ‘fallers’ [mean variability 5%] [33].

#### Baseline associations

**Fig. 1:**
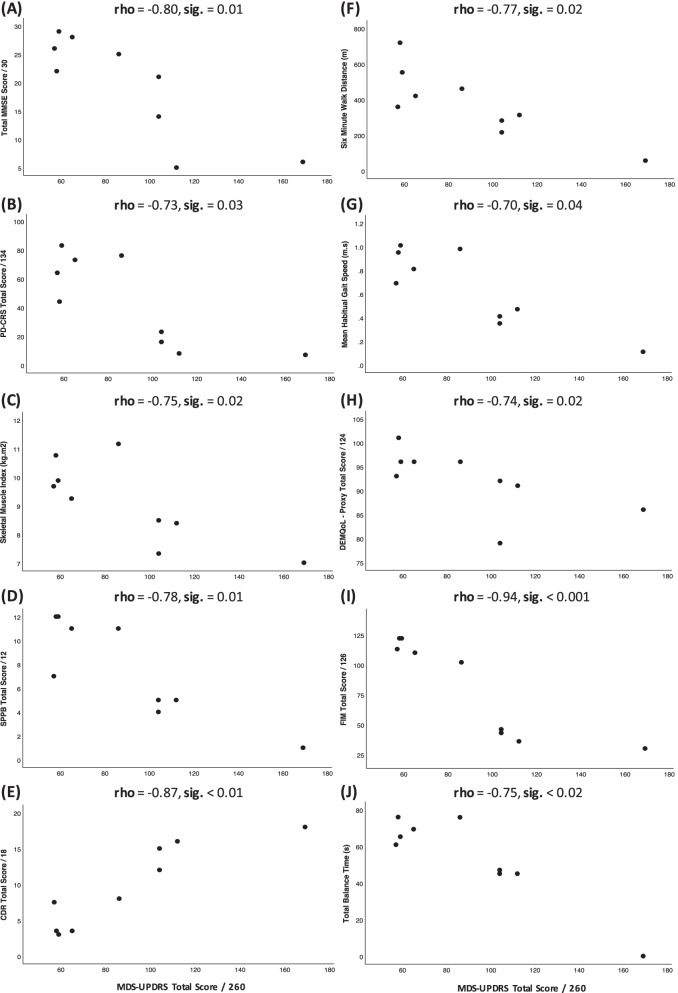
Significant Baseline Associations between Functional Independence and Clinical Characteristics. *Note: A line of best fit was not appropriate, as correlation analysis was performed on ranks (Spearman) not the raw data.* Higher scores in the MDS-UPDRS [21], and lower scores on the FIM [25], MMSE [26], PD-CRS [27], SPPB [34], and DEMQoL [35] indicate worse performance on that measure respectively. Rho = Spearman’s rank correlation coefficient, CDR = Clinical Dementia Rating [29], DEMQoL – Proxy = Dementia Quality of Life Scale – proxy, [35] SPPB = Short Physical Performance Battery, MDS-UPDRS = Movement Disorder Society Unified Parkinson’s Disease Rating Scale, FIM = Functional independence measure, MMSE = Mini-mental state exam, PD-CRS = Parkinson’s disease Cognitive rating scale, kgm.^−2^ = kilogram/metre squared, m.s = metres per second, m = metre, s = seconds. *Total Balance time* is the time held in each of 6 positions [18], adding each successful attempts (15 s/position) and the time spent in the last/failed position (≤ 15 s)

Greater functional independence (lower MDS-UPDRS total score) was significantly inversely associated with greater physical function, balance, cognition, quality of life, muscle mass ratio, walking endurance, habitual walking speed and cadence, more short walking bouts (5–10 s), and a lower overall dementia severity (CDR) (*p* < 0.05 for all, Fig. 1), as hypothesized. Unexpectedly, total functional independence was not significantly associated with maximal grip or leg strength, nor maximal walking speed, physical activity or walking volumes, gait parameters or nutritional status (p ≥ 0.05, Supplementary Text S1, Additional File 1). However, higher (worse) scores on the motor and non-motor experiences sub-scale (part I/II of the MDS-UPDRS) *were* significantly associated with lower daily physical activity, walking time and daily walking bouts of any duration (*p* < 0.05). There were no associations with any accelerometry-derived measures of gait quality of known clinical relevance.

Lastly, there were several important associations observed in secondary outcomes. Higher physical activity was positively associated with better cognition, and both were associated with a range of other secondary outcomes of interest including functional independence, quality of life, disease status and functional measures (*p* < 0.05) (Supplementary Table S5, Additional File 1).

### Wait-list period

Although of borderline statistical significance (*p* = 0.051), functional independence tended to decline during this period (Table 2). Seven participants, including three who did not complete the subsequent exercise intervention per protocol, experienced clinically meaningful worsening (higher total score in the MDS-UPDRS) which exceeded the annual worsening in disease severity of 4.7 points for PD cohorts in only 8 weeks [23].

**Table 2:** Changes in functional independence and key outcomes over the 8-week wait-list period

	n	Baseline Median (range)	Pre—Intervention Median (range)	W^a^	Sig	Median change score (95% Hodges-Lehmann CI)
Functional Independence						
MDS-UPDRS total /260	9	86 (57–169)	106 (45–180)	1.995	0.051	13 (-1, 34.5)
Exercise completers^a^	6	62 (57–169)	78.5 (45–180)	1.363	0.173	6.5 (-10, 19.5)
Part I/II sub-score /104	9	36 (16–75)	38 (13–84)	1.897	0.058	6 (-0.5, 14)
Part III sub-score /132	9	46 (33–82)	55 (32–95)	1.186	0.236	5.5 (-6.5, 17.5)
Part IV /24	9	0 (0–12)	2 (0–8)	0.841	0.4	0.5 (-1.5, 3)
FIM total score /126	9	102 (30–122)	100 (25–121)	-0.889	0.374	-3 (-11, 9)
Strength & Physical Function						
Leg press 1RM, N	6	1375 (355–3100)	1200 (830–3000)	-1.682	0.093	-150 (-373, 40)
Leg extension 1RM, N·m	6	240 (35–550)	180 (120–570)	-1.572	0.116	-65 (-150, 35)
Triceps extension 1RM, N	6	450 (150–850)	470 (100–800)	-0.946	0.344	-45 (-200, 40)
SPPB total score, /12	9	7 (1–12)	7 (0–12)	-2.414	0.016	-1.5 (-2.5, -0.5)
Sub-scores:						
Balance, /4		4 (0–4)	2 (0–4)	-1.414	0.157	0 (-1, 0)
Gait, /4		3 (1 -4)	2 (0–4)	-1.134	0.257	-0.5 (-1, 0.5)
Sit-to-stand, /4		2 (0–4)	1 (0–4)	-1.633	0.102	-1 (-2, 0)
Habitual gait speed, ms^−1^	7	0.69 (.11–1.01)	0.75 (0.42–1.09)	-0.169	0.866	-0.1 (-0.19, 0.13)
Maximal gait speed, ms^−1^	7	1.41 (0.75–2.03)	1.21 (0.94–2.38)	-0.338	0.735	-0.03 (-0.37, 0.29)
Five time sit-to-stand, s	7	13.8 (8.1–33.7)	11.5 (8.09–28.3)	-0.169	0.866	-0.2 (-3.8, 3.3)
Total balance time, s	9	60.84 (0.0–75.91)	47.72 (0.0–83.43)	-1.183	0.237	-1.9 (-22.5, 8.7)
Average grip strength, kg	8	25.5 (12.5–40.0)	23.75 (7–41.50)	-0.17	0.865	0 (-3.75, 2.75)
Six-minute walk, m	8	358 (57–718)	353 (0–727)	-0.7	0.484	-14 (-115, 44)
Physical activity						
Total daily activity, g/hr	9	120.1 (45.4–262.7)	91.0 (34.7–186.6)	-2.429	0.015	-20.9 (-48.4, -7.2)
Proportion of day, %		10.81 (7.81–20.29)	9.56 (1.67–17.2)	-2.31	0.021	-2.56 (-4.90, -0.56)
Daily walking time, mins		46.8 (7.2–172.2)	34.8 (4.8–139.2)	-2.666	0.008	-16.4 (-29.9. -4.0)
Proportion of day, %		3.25 (0.49–11.94)	2.43 (0.33–9.68)	-2.666	0.008	-1.1 (-2.08, -0.28)
Step count, n		4158 (567–12,511)	2710 (400–9327)	-2.31	0.021	-998 (-2200, -113)
Total bouts of walking, n		139 (35–377)	113 (21–317)	-2.433	0.015	-26 (-57, -4)
Bout lengths						
5 to 10 s, n		66 (21–180)	53 (13–173)	-2.106	0.044	-9 (-18.25, 0.00)
10 to 20 s, n		47 (12–106)	40 (6–79)	-2.138	0.033	-9 (-17.5, -0.50)
20 to 30 s, n		17 (2–27)	10 (2–28)	-1.606	0.108	-2.5 (-5.5, 0.5)
30 to 60 s, n		13 (0–39)	8 (0–23)	-2.136	0.033	-4.5 (-9.5, 0)
60 to 120 s, n		3 (0–14)	1 (0–11)	-1.807	0.071	-1.5 (-2.5, 0)
≥ 120 s, n		1 (0–11)	0 (0–6)	-1.89	0.059	-0.5 (-2.5, 0)
Cognition						
PD-CRS total score /134	9	44 (7–83)	42 (10 -77)	-0.422	0.673	-1 (-6, 3)
Posterior-cortical /30		26 (1–28)	26 (4–29)	0.647	0.518	0.5 (-0.5, 2)
Fronto-cortical /104		18 (1 -55)	17 (6–48)	-0.831	0.406	-1.5 (-5, 2)
MMSE total score /30	9	22 (5–29)	17 (3–26)	-1.916	0.055	-3 (-4, 0)

Significance values where α < 0.05 are bolded. *MDS-UPDRS* Movement Disorder Society Unified Parkinson’s Disease Rating Scale [21]. Total score includes parts I-IV, Part III is the assessor rated motor score. ^a^ Exercise completers includes only the scores of participants who completed the subsequent exercise intervention as per protocol for analysis of the primary outcome. All values a presented as median (range). Higher scores in the MDS-UPDRS; and lower scores on the FIM, MMSE, and PD-CRS indicate worse performance on that measure respectively. *1RM* One repetition maximum lift, *FIM* Functional independence measure [25], *MMSE* Mini-mental state exam [26], *PD-CRS* Parkinson’s disease Cognitive rating scale [27], *SPPB* Short Physical Performance Battery [34], *kg* kilogram, *kgm*^−1^ kg/metre, *s* second, *cm* centimeter, *N* Newton, N·m Newton metre.^a^ W Wilcoxon Signed Rank standardized test statistic

**Table 3:** Changes in functional independence and key outcomes over the 8-week exercise intervention

	n	Pre – Intervention Median (range)	Post-Intervention Median (range)	W^a^	Sig	Median change score (95% Hodges-Lehmann CI)
Functional Independence						
MDS-UPDRS total /260	6	78.5 (45–180)	72.5 (33–157)	-2.207	0.027	-8 ( -17.5, -2)
Part I/II sub-score /104		24.5 (13–84)	30.5 (10–69)	-1.153	0.074	-3.5 (-10.5, 6)
Part III sub-score / 132		53 (32–88)	41 (23–79)	-1.153	0.249	-7.5 (-12, 1)
Part IV /24		1 (0–8)	1 (0–9)	1.00	0.317	0 (0, 0.5)
FIM total score /126	6	107 (25–121)	109.5 (19–122)	0.315	0.752	1 (-5, 5.5)
Strength & Physical Function						
Leg press 1RM, N	5	1250 (1080–3000)	2000 (1800–3350)	2.023	0.043	600 (350, 850)
Leg extension 1RM, N·m	5	210 (140–570)	330 (280–720)	2.023	0.043	137 (120, 160)
Triceps extension 1RM, N		-	-	-	-	-
SPPB total score, /12	6	8 (0–12)	12 (0–12)	1.857	0.063	2 (0, 4)
Sub-scores:						
Balance, /4		4 (0–4)	4 (0–4)	1	0.317	0 (0, 1)
Gait, /4		3.5 (0—4)	4 (0–4)	1.414	0.157	0.5 (0, 1)
Sit-to-stand, /4		1.5 (0–4)	4 (0–4)	1.841	0.068	1 (0, 3)
Habitual gait speed, ms^−1^	5	0.83 (0.5–1.09)	1.01 (0.61–1.39)	1.826	0.068	0.15 (0, 0.3)
Maximal gait speed, ms^−1^	5	1.5 (0.94–2.38)	1.83 (1.07–2.25)	1.625	0.104	0.19 (-0.13, 0.71)
Five time sit-to-stand, s	5	11.5 (8.1–19.9)	9.6 (6.7–14.9)	-2.023	0.043	-3.0 (-5.0, -1.0)
Total balance time, s	6	61.5 (0.0–83.4)	65.5 (0.0–84.3)	2.203	0.043	3.0 (0.4, 7.9)
Average grip strength, kg		-	-	-	-	-
Six-minute walk, m	5	505 (217–727)	458 (255–789)	0.674	0.5	44 (-63, 62)
Physical activity						
Total daily activity, g/hr	6	116.7 (34.7–186.6)	118.7 (37.4–197.4)	0.105	0.917	1.99 (-12.7, 17.4)
Proportion of day, %		10.3 (4.75–17.2)	11.5 (4.01–16.9)	0.524	0.6	0.3 (-0.8, 1.5)
Daily walking time, mins		49.5 (6.6–139.2)	51 (1.8–160.2)	1.153	0.249	6.96 (-10.3, 18.5)
Proportion of day, %		3.45 (0.45–9.68)	3.53 (0.11–11.11)	1.153	0.249	0.5 (-0.7, 1.3)
Step count, n		4617 (455–9327)	4884 (80–11,772)	0.734	0.463	528 (-1889, 2632)
Total bouts of walking, n		163.5 (29.5–317)	190 (5–341)	1.153	0.249	23 (-3.25, 29.5)
Bout lengths						
5 to 10 s, n		89 (19–173)	92 (3–164)	-0.105	0.917	-1 (-12.5, 10)
10 to 20 s, n		47.5 (7–79)	66.5 (2–98)	1.782	0.075	11 (-2, 21.5)
20 to 30 s, n		13.5 (2.5–28)	16.5 (0–26)	0.105	0.916	0.25 (-2.25, 5)
30 to 60 s, n		8 (1–23)	10 (0–33)	1.393	0.173	3.5 (-2, 9.5)
60 to 120 s, n		2 (0–11)	2.5 (0–12)	0.68	0.496	1 (-1.5, 2)
≥ 120 s, n		0.5 (0–6)	0 (0–10)	0.272	0.785	0 (-2, 2.5)
Cognition						
PD-CRS total score /134	6	62.5 (10–77)	68.5 (16–102)	2.207	0.027	8 (4, 17.5)
Posterior-cortical /30		26.5 (4–29)	29 (9 -30)	1.841	0.066	2 (0, 4)
Fronto-cortical /104		36 (6–48)	40 (7–73)	1.892	0.058	6.5 (0, 17)
MMSE total score /30	6	21.5 (3–26)	25 (11–29)	2.207	0.027	4.5 (1.5, 7.5)

Significance values where α < 0.05 are bolded. MDS-UPDRS Movement Disorder Society Unified Parkinson’s Disease Rating Scale [21]. Total score includes parts I-IV, Part III is the assessor rated motor score. a Exercise completers includes only the scores of participants who completed the subsequent exercise intervention as per protocol for analysis of the primary outcome. All values a presented as median (range). Higher scores in the MDS-UPDRS; and lower scores on the FIM, MMSE, and PD-CRS indicate worse performance on that measure respectively. 1RM One repetition maximum lift, FIM Functional independence measure [25], MMSE Mini-mental state exam [26], PD-CRS Parkinson’s disease Cognitive rating scale [27], SPPB Short Physical Performance Battery [34], kg kilogram, kgm−1 kg/metre, s second, cm centimeter, N Newton, N·m Newton metre. a W Wilcoxon Signed Rank standardized test statistic

There were no significant changes in the FIM (*p* = 0.374, Table 2) or any measures of maximal strength or balance during this period (p ≥ 0.05). However, global measures of physical function (SPPB, Short Physical Performance Battery) [34] significantly worsened (lower score) with eight participants declining by equal to, or more than the 1 point MCID for the SPPB [36] (*p* = 0.016).

Additionally, physical activity significantly decreased during the wait-list period (*p* = 0.015) as did step count, with a median decrease of 998 steps/day (*p* = 0.021, Table 2). Decline in cognition as measured by the MMSE (*p* = 0.055) and PD-CRS (*p* = 0.673) was not significant, however all but one participant declined in the MMSE by an amount greater than or equal to the 2 – 4 points annual observed annual worsening in LBD [5].

Overall, nutritional status (Mini-Nutritional Assessment – Short Form) [37] significantly worsened during the wait-list period with four participants transitioning from ‘at-risk’ to ‘malnourished’ classification (*p* = 0.046, Supplement Table S6, Additional File 1). Fat-free mass significantly decreased in all participants (*p* = 0.011, -0.26 to -2.97 kg), body mass decreased in seven (2.5 to 8.5 kg), and of those seven, five lost more than the clinically significant 4.5 kg annual loss of mass (a criterion for frailty) [38].

Lastly, there were no significant changes in psychosocial and quality of life measures, and only two significant changes to gait quality metrics indicative of variability of other metrics, which were of unclear clinical significance (p ≥ 0.05, Supplement Table S6, S8, and S9).

### Intervention period

**Fig. 2:**
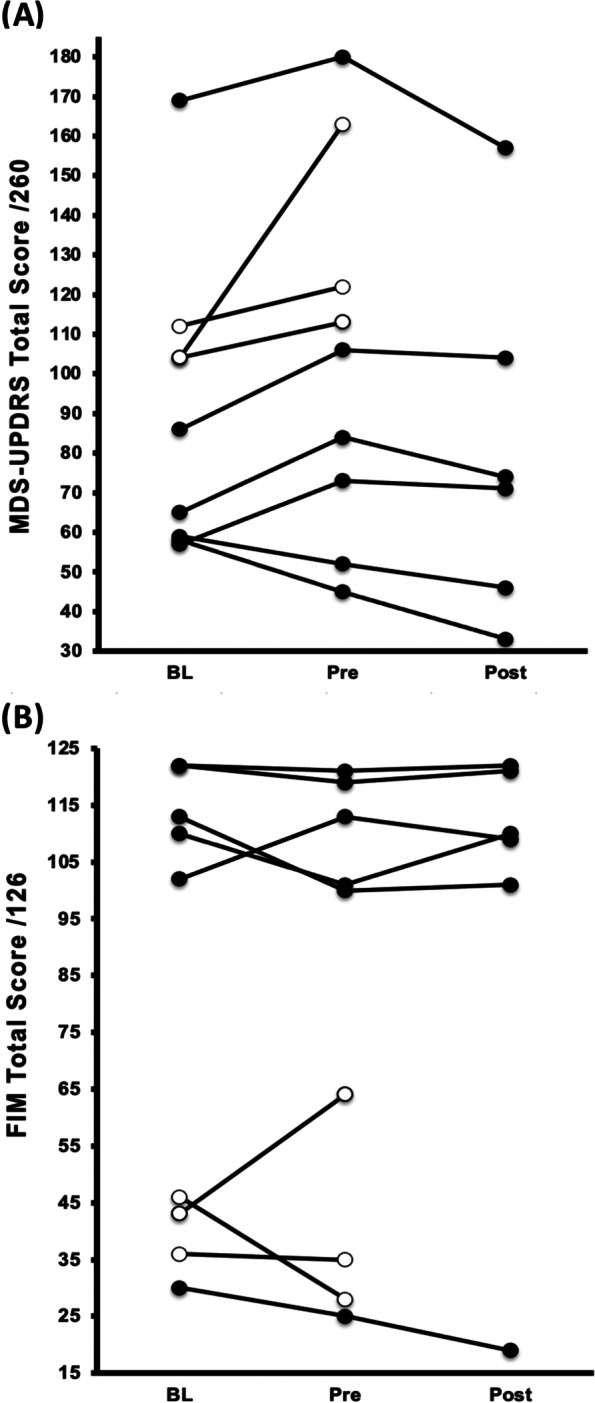
Individual Changes in Functional Independence across All Time Points. **A** MDS-UPDRS Movement Disorder Society Unified Parkinson’s Disease Rating Scale total score [21], and **B** FIM Functional independence Measure [25]. White-filled circles indicated participants who completed wait-list period but did not complete exercise intervention per protocol. The intervention results of one participants are documented seperately in a case report due to an extended wait-list period before commencing exercise [24]

Six participants completed the trial (*median* 23 sessions attended, *range* 19 – 24 of 24), for 136 h of intervention (*mean* 22.6 h/participant). Adherence to the training ranged from 79 – 100% of all offered sessions. The three participants who did not complete the intervention were generally frailer with poor disease status (Fig. 2). Results are shown in Table 3.

There was a significant improvement (lower score) in functional independence (MDS-UPDRS) for all participants, with four participants improving by an amount equivalent to reversing the expected annual decline with disease progression (Fig. 2) [23]. Additionally, there were significant improvements in physical function and strength, including sit-to-stand, total balance time (longer time), and maximal strength on the bilateral leg press and leg extension (*p* = 0.043 for all, Table 3). There was no significant change in the FIM (*p* = 0.752) or physical activity volume outside of the exercise sessions (*p* = 0.917).

As hypothesized, cognition improved significantly in all participants for the MMSE and PD-CRS, with five improving equal to, or exceeding the typical annual decline in LBD (2 – 4 points) [5], and two improving more than the MCID (10 points) for the PD-CRS [39].

Nutritional status, body composition, psychosocial and quality of life measures did not change significantly following the brief exercise intervention (p ≥ 0.05 for all, Supplementary Table S7, Additional File 1). There were a few accelerometry-derived gait metrics that significantly changed following intervention (Supplementary Table S8, S10, Additional File 1). Step time increased for all participants without changes to cadence or stride length, which may indicate a slight slowing of gait, however it was highly variable and of unclear clinical significance due to the known fluctuations in similar PD cohorts [40]. Similarly, there was a significant decrease in medio-lateral symmetry during stepping which could indicate that gait became slightly less regular, however there are currently no clinical data to evaluate the clinical relevance of this change.

## Discussion

The PRIDE study was the first trial to evaluate the short-term trajectory and the effects of a tailored, progressive, high-intensity exercise intervention on functional independence and its correlates in older adults with LBD.

The characteristics of participants within PRIDE varied, including both mild-to-moderately impaired individuals living in the community, as well as those with severe dementia in residential aged care. Therefore, our cohort included participants with a greater range of disease severity than other cross-sectional LBD cohorts evaluating similar metrics [6]. The participants had greater disease severity, motor impairment, frailty and disease burden than similar cohorts described in the literature [10, 41, 42]. However, our sample was predominantly male and white non-Hispanic, which may not be representative of the demographic characteristics of the broader LBD community [43]. Taken together, these findings may explain some of the associations we observed in the PRIDE cohort, however we acknowledge that due to the small sample, our results should be interpreted cautiously and need further investigation in larger and more diverse, controlled trials.

Our first key finding was that functional independence was associated with better balance, greater physical function, walking endurance, cognition, quality of life and lower dementia severity, which is consistent with the literature [6, 44]. Importantly, all these characteristics are known to be amenable to targeted exercise interventions [17]. Unexpectedly, functional independence was not directly associated with maximal strength as hypothesized. However, strength *was* found to be strongly associated with cognition, which is promising, as in similar cohorts cognition was found to be associated with functional independence and mediated by improvements in strength following progressive, high-intensity anabolic exercise [6, 45]. Thus, these findings provide new insight into the relationships between important clinical characteristics which may be amenable to exercise intervention and functional independence.

Our second key finding was that during a relatively brief wait-list period, participants became markedly frailer, more cognitively impaired, and less independent. Although the participants did not decline in maximal measures of strength, the study found clinically significant decreases in several indicators of frailty in most participants including physical activity, physical function, muscle mass, weight and nutritional status. This occurred alongside clinically significant worsening of cognition and functional independence, which suggests that frailty itself may play a role in the rapid and fluctuating disease course observed in LBD [5]. For example, the prevalence of frailty in LBD and its contributing factors such as polypharmacy, delirium, malnutrition, and lower physical activity are significantly higher than in AD [6–, 7–10] and are strong predictors of disease trajectory [5]. Furthermore, the prevalence of sarcopenia and frailty increases with dementia severity [46], and can be exacerbated by even short periods of inactivity and bed-rest [15]. Conversely, high levels of physical activity and progressive, high-intensity exercise in these cohorts are protective against frailty and sarcopenia, loss of lean mass, and cognitive decline [15]. This is the first time to our knowledge that indices of frailty have been documented longitudinally in LBD, albeit over a short period of time and in a small sample. These observations provide further insight to the potential contribution of frailty to the disease trajectory and treatment targets within LBD.

Our most important key finding was that the application of 8 weeks of progressive, high-intensity anabolic exercise targeting strength and balance in those with LBD stabilised clinical status and improved functional independence, confirming our second hypothesis. High intensity, progressive exercise was well tolerated (training intensity ≥ 80% maximum strength), had high compliance (≥ 80% of sessions attended), and appears safe (only one related adverse event in 136 h of training). Functional independence (MDS-UPDRS) improved significantly following exercise in all participants, with four of six participants improving by an amount exceeding the annual deterioration of 4.7 points observed in PD [23], thus essentially erasing a year’s worth of LBD progression. While this finding is promising, it is unclear if this reflects a true change in functional independence or, as the scale was originally designed for, a change in disease severity as the secondary outcome measure of functional independence, the FIM, did not change significantly throughout the study. We had previously theorised that the FIM, predominantly used in inpatient rehabilitation [31, 47, 48] may not be sensitive enough to interventional changes specific to LBD, and thus decided to include the MDS-UPDRS concurrently as the main measure of functional independence.

Additionally, there were also significant improvements in strength, physical function, and cognition following exercise, with many participants experiencing clinically significant changes. Measures of nutritional status, body composition and physical activity did change significantly in either direction after the brief period of training, which may indicate that anabolic adaptations for these outcomes may require longer intervention periods. For example, a similar study in older, frail adults with a high prevalence of cognitive impairment reported significant improvements in physical activity following 10 weeks of PRT [49]. Likewise, that study, as well as other PRT exercise programs in PD of 12 weeks duration, reported significant improvements in muscle mass [50].

### Limitations

The validity of observations made in PRIDE is limited by a small, heterogenous sample, the inability of some participants to complete all assessments due to fluctuating cognition, and reduced sample for exercise intervention. First, not blinding the assessor/interventionalist due to limited study resources and the cross-over design may have introduced observer and social desirability bias in the assessments.

Second, the interpretation of significant baseline associations and change scores was limited to non-parametric statistics, as it was not appropriate to perform multiple linear regressions with the small sample size. This limited our ability to control for covariates such as age, sex and education, which have well-known influences on outcomes such as cognition, strength, physical function, exercise capacity, and muscle mass [51, 52]. Additionally, the evaluation of a comprehensive assessment battery with no multiple comparison statistical correction increased the risk of type I error, while evaluating a small, ample increases the risk of a type II error due to low power. In particular, we did not interpret a *p* value of > 0.05 when the power was < 0.8 as proving that there was no effect, but rather that there was *no evidence* of an effect either way. Notably, a small sample size does not introduce the possibility a type II error if the *p* value *is* actually < 0.05, so we are confident in the significant differences that we did find. Additionally, the clinical meaningfulness of change scores were also provided to the reader where appropriate to better inform the interpretation of changes scores regardless of the statistical significance.

Third, LBD is a disease characterised by fluctuating cognition and function, more so than observed in other dementias [5]. In addition to the inherent limitations of all cross-sectional and longitudinal study designs relating to point estimates of an outcome [53], the repeatability of measurements we captured during our baseline assessment is likely to be reduced due to this disease variability. For example, the clinically significant deterioration in cognition observed during the wait-list period, followed by the equally large improvement following exercise must be interpreted cautiously as daily fluctuations in cognition for individuals with LBD have yet to be characterised. Conversely, the baseline control crossover design for this short intervention appeared advantageous as participants were compared to their own baseline scores, which provided a somewhat controlled observation of changes over time in a diverse, fluctuating group.

Lastly, while intense exercise was well adhered to and anecdotally well-tolerated by participants with only one minor adverse outcome, our study did not collect qualitative data on the acceptability and experiences of the intervention. Thus, the relationships observed in this study and feasibility of intervention must be interpreted with appropriate caution, and primarily used to guide future, more robust investigations in LBD.

## Conclusion

The PRIDE trial was the first exercise trial to evaluate the effect of exercise specifically on individuals diagnosed with LBD. This trial provides important insight for the design of larger, higher quality trials for further evaluation of non-pharmacological treatments such as progressive, high-intensity exercise as a viable treatment for the aggressive, rapidly progressing disease of Lewy body dementia.

## Supplementary Information

**Additional file 1: Supplementary Text (S1).** detailing Additional Methodology & Results information; **Supplementary Tables (S1-S10).** detailing the Interpretation of Accelerometry-derived Physical Activity, Gait; Baseline Characteristics of Frailty and Sarcopenia; Accelerometry-derived Gait and Physical Activity Variables; Accelerometry-derived Measures of Gait Quality; Physical Activity and Cognition Associations with Clinical Characteristics at Baseline; Change in Clinical Characteristics during Wait-list period; Change in Clinical Characteristics during Intervention period; Axivity-derived Gait Quality and Quantity Change during Wait-list and Intervention Period; Wait-list Period Non-significant Changes in Accelerometry-derived Gait Quality; and Intervention Period Non-Significant Changes in Accelerometry-derived Gait Quality. This is followed by figures including (in order): and **Supplementary Figures (S1 & S2).** detailing Consort Flow Diagram; and Average 24-hour activity breakdown – Baseline.

## Data Availability

The datasets generated and/or analysed during the current study are not publicly available currently due to considerations about potential patient identification in a small sample, but are available from the corresponding author on reasonable request.

## Abbreviations

PRIDE: Promoting Independence in Lewy Body Dementia through Exercise
LBD: Lewy body dementia
PRT: Progressive resistance training
PDD: Parkinson’s disease dementia
PD: Parkinson’s disease
AD: Alzheimer’s disease dementia
HREC: Human Research Ethics Committee
GP: General Practioners
AEP: Accredited exercise physiologist
MDS-UPDRS: Movement Disorder Society Unified Parkinson’s Disease Rating Scale
MCID: Minimum clinically important difference
FIM: Functional Independence Measure
MMSE: Mini-mental State Examination
PD-CRS: Parkinson’s Disease Cognitive Rating Scale
CDR: Clinical Dementia Rating
SPPB: Short Physical Performance Battery

## References

[1] McKeith IG, Boeve BF, Dickson DW, Halliday G, Taylor J-P, Weintraub D (2017). Diagnosis and management of dementia with Lewy bodies: Fourth consensus report of the DLB Consortium. Neurology.

[2] Jones SV, O'brien J (2014). The prevalence and incidence of dementia with Lewy bodies: a systematic review of population and clinical studies. Psychol Med.

[3] Kane JP, Surendranathan A, Bentley A, Barker SA, Taylor J-P, Thomas AJ (2018). Clinical prevalence of Lewy body dementia. Alzheimer's research & therapy.

[4] World Health Organization. Global action plan on the public health response to dementia 2017–2025. [Internet] Geneva: World Health Organisation; 2017. Available from: https://apps.who.int/iris/bitstream/handle/10665/259615/?sequence=1.

[5] Mueller C, Ballard C, Corbett A, Aarsland D (2017). The prognosis of dementia with Lewy bodies. The Lancet Neurology.

[6] Mc Ardle R, Del Din S, Donaghy P, Galna B, Thomas A, Rochester L (2020). Factors that influence habitual activity in mild cognitive impairment and dementia. Gerontology.

[7] Allan LM, Ballard CG, Rowan EN, Kenny RA (2009). Incidence and prediction of falls in dementia: a prospective study in older people. PloS one.

[8] FitzGerald JM, Perera G, Chang-Tave A, Price A, Rajkumar AP, Bhattarai M (2019). The incidence of recorded delirium episodes before and after dementia diagnosis: differences between dementia with Lewy bodies and Alzheimer's disease. J Am Med Dir Assoc.

[9] Roque M, Salva A, Vellas B (2013). Malnutrition in community-dwelling adults with dementia (NutriAlz Trial). J Nutr Health Aging.

[10] Borda MG, Soennesyn H, Steves CJ, Vik-Mo AO, Pérez-Zepeda MU, Aarsland D (2019). Frailty in Older Adults with Mild Dementia: Dementia with Lewy Bodies and Alzheimer’s Disease. Dementia and Geriatric Cognitive Disorders Extra.

[11] Mueller C, Soysal P, Rongve A, Isik AT, Thompson T, Maggi S (2019). Survival time and differences between dementia with Lewy bodies and Alzheimer’s disease following diagnosis: A meta-analysis of longitudinal studies. Ageing Res Rev.

[12] Inskip M, Mavros Y, Sachdev PS, Fiatarone Singh MA (2016). Exercise for individuals with Lewy body dementia: a systematic review. PLoS ONE.

[13] Connors MH, Quinto L, McKeith I, Brodaty H, Allan L, Bamford C (2018). Non-pharmacological interventions for Lewy body dementia: a systematic review. Psychol Med.

[14] Taylor J-P, McKeith IG, Burn DJ, Boeve BF, Weintraub D, Bamford C (2020). New evidence on the management of Lewy body dementia. The Lancet Neurology.

[15] Dent E, Lien C, Lim WS, Wong WC, Wong CH, Ng TP (2017). The Asia-Pacific clinical practice guidelines for the management of frailty. J Am Med Dir Assoc.

[16] Prodoehl J, Rafferty MR, David FJ, Poon C, Vaillancourt DE, Comella CL (2015). Two-year exercise program improves physical function in Parkinson’s disease: the PRET-PD randomized clinical trial. Neurorehabil Neural Repair.

[17] Heyn P, Abreu BC, Ottenbacher KJ (2004). The effects of exercise training on elderly persons with cognitive impairment and dementia: a meta-analysis. Arch Phys Med Rehabil.

[18] Inskip M, Mavros Y, Sachdev PS, Singh MAF (2019). Promoting independence in Lewy body dementia through exercise (PRIDE) study: Protocol for a pilot study. Contemporary clinical trials communications.

[19] Australian and New Zealand Clinical Trials Registry [Internet]: Sydney (NSW): NHMRC Clinical Trials Centre, University of Sydney (Australia); 2016 - Identifier ACTRN12616000466448. The PRIDE trial: Promoting Independence in Lewy Body Dementia through Exercise: 2016 Apr 04 [cited 2022 Jan 01]. Available from: https://www.anzctr.org.au/Trial/Registration/TrialReview.aspx?id=370405.

[20] Eldridge SM, Chan CL, Campbell MJ, Bond CM, Hopewell S, Thabane L (2016). CONSORT 2010 statement: extension to randomised pilot and feasibility trials. Pilot Feasibil Stud.

[21] Goetz CG, Tilley BC, Shaftman SR, Stebbins GT, Fahn S, Martinez-Martin P (2008). Movement Disorder Society-sponsored revision of the Unified Parkinson's Disease Rating Scale (MDS-UPDRS): scale presentation and clinimetric testing results. Movement Dis.

[22] Galvin JE (2015). Improving the clinical detection of Lewy body dementia with the Lewy body composite risk score. Alzheimer's & Dementia: Diagnosis, Assessment & Disease Monitoring.

[23] Holden SK, Finseth T, Sillau SH, Berman BD (2018). Progression of MDS-UPDRS scores over five years in de novo Parkinson disease from the Parkinson's progression markers initiative cohort. Movement Dis Clin Pract.

[24] Inskip M, Mavros Y, Sachdev PS, Singh MAF (2020). Interrupting the trajectory of frailty in dementia with Lewy bodies with anabolic exercise, dietary intervention and deprescribing of hazardous medications. BMJ Case Rep CP.

[25] Linacre JM, Heinemann AW, Wright BD, Granger CV, Hamilton BB (1994). The structure and stability of the Functional Independence Measure. Arch Phys Med Rehabil.

[26] Folstein MF, Folstein SE, McHugh PR (1975). “Mini-mental state”: a practical method for grading the cognitive state of patients for the clinician. J Psychiatr Res.

[27] Pagonabarraga J, Kulisevsky J, Llebaria G, García-Sánchez C, Pascual-Sedano B, Gironell A (2008). Parkinson's disease-cognitive rating scale: a new cognitive scale specific for Parkinson's disease. Movement Dis.

[28] Brink TL, Yesavage JA, Lum O, Heersema PH, Adey M, Rose TL (1982). Screening tests for geriatric depression. Clin Gerontol.

[29] Morris JC (1997). Clinical dementia rating: a reliable and valid diagnostic and staging measure for dementia of the Alzheimer type. Int Psychogeriatr.

[30] Boustani M, Campbell N, Munger S, Maidment I, Fox C. Impact of anticholinergics on the aging brain: a review and practical application. 2008.

[31] Ellis T, Katz DI, White DK, DePiero TJ, Hohler AD, Saint-Hilaire M (2008). Effectiveness of an inpatient multidisciplinary rehabilitation program for people with Parkinson disease. Phys Ther.

[32] Cruz-Jentoft AJ, Baeyens JP, Bauer JM, Boirie Y, Cederholm T, Landi F (2010). Sarcopenia: European consensus on definition and diagnosisReport of the European Working Group on Sarcopenia in Older PeopleA. J. Cruz-Gentoft. Age Ageing..

[33] Hausdorff  JM (2009). Gait dynamics in Parkinson’s disease: common and distinct behavior among stride length, gait variability, and fractal-like scaling. Chaos.

[34] Guralnik JM, Simonsick EM, Ferrucci L, Glynn RJ, Berkman LF, Blazer DG (1994). A short physical performance battery assessing lower extremity function: association with self-reported disability and prediction of mortality and nursing home admission. J Gerontol.

[35] Smith S, Lamping D, Banerjee S, Harwood R, Foley B, Smith P (2005). Measurement of health-related quality of life for people with dementia: development of a new instrument (DEMQOL) and an evaluation of current methodology. Health Technol Assessment (Winchester, England).

[36] Kwon S, Perera S, Pahor M, Katula J, King A, Groessl E (2009). What is a meaningful change in physical performance? Findings from a clinical trial in older adults (the LIFE-P study). JNHA-J Nutr Health Aging.

[37] Kaiser MJ, Bauer JM, Ramsch C, Uter W, Guigoz Y, Cederholm T (2009). Validation of the Mini Nutritional Assessment Short-Form (MNA®-SF): A practical tool for identification of nutritional status. JNHA-J Nutr Health Aging.

[38] Fried LP, Tangen CM, Walston J, Newman AB, Hirsch C, Gottdiener J (2001). Frailty in older adults: evidence for a phenotype. J Gerontol A Biol Sci Med Sci.

[39] Fernández de Bobadilla R, Pagonabarraga J, Martínez‐Horta S, Pascual‐Sedano B, Campolongo A, Kulisevsky J (2013). Parkinson's disease‐cognitive rating scale: Psychometrics for mild cognitive impairment. Movement Disord.

[40] Del Din S, Godfrey A, Galna B, Lord S, Rochester L (2016). Free-living gait characteristics in ageing and Parkinson’s disease: impact of environment and ambulatory bout length. J Neuroeng Rehabil.

[41] Galperin I, Hillel I, Del Din S, Bekkers EM, Nieuwboer A, Abbruzzese G (2019). Associations between daily-living physical activity and laboratory-based assessments of motor severity in patients with falls and Parkinson's disease. Parkinsonism Relat Disord.

[42] Fereshtehnejad S-M, Damangir S, Cermakova P, Aarsland D, Eriksdotter M, Religa D (2014). Comorbidity profile in dementia with Lewy bodies versus Alzheimer’s disease: a linkage study between the Swedish Dementia Registry and the Swedish National Patient Registry. Alzheimer's research & therapy.

[43] Hou CE, Yaffe K, Pérez-Stable EJ, Miller BL (2006). Frequency of dementia etiologies in four ethnic groups. Dement Geriatr Cogn Disord.

[44] Perrault A, Wolfson C, Egan M, Rockwood K, Hogan DB (2002). Prognostic factors for functional independence in older adults with mild dementia: results from the Canadian study of health and aging. Alzheimer Dis Assoc Disord.

[45] Mavros Y, Gates N, Wilson GC, Jain N, Meiklejohn J, Brodaty H (2017). Mediation of cognitive function improvements by strength gains after resistance training in older adults with mild cognitive impairment: outcomes of the study of mental and resistance training. J Am Geriatr Soc.

[46] Ogawa Y, Kaneko Y, Sato T, Shimizu S, Kanetaka H, Hanyu H (2018). Sarcopenia and muscle functions at various stages of Alzheimer disease. Front Neurol.

[47] Dodds TA, Martin DP, Stolov WC, Deyo RA (1993). A validation of the functional independence measurement and its performance among rehabilitation inpatients. Arch Phys Med Rehabil.

[48] Cournan M (2011). Use of the functional independence measure for outcomes measurement in acute inpatient rehabilitation. Rehabil Nurs.

[49] Fiatarone MA, O'Neill EF, Ryan ND, Clements KM, Solares GR, Nelson ME (1994). Exercise training and nutritional supplementation for physical frailty in very elderly people. N Engl J Med.

[50] Dibble LE, Hale TF, Marcus RL, Droge J, Gerber JP, LaStayo PC (2006). High-intensity resistance training amplifies muscle hypertrophy and functional gains in persons with Parkinson's disease. Movement Dis.

[51] Doherty TJ (2001). The influence of aging and sex on skeletal muscle mass and strength. Curr Opin Clin Nutr Metab Care.

[52] Van Hooren S, Valentijn A, Bosma H, Ponds R, Van Boxtel M, Jolles J (2007). Cognitive functioning in healthy older adults aged 64–81: a cohort study into the effects of age, sex, and education. Aging Neuropsychol Cogn.

[53] Hofer SM, Sliwinski MJ, Flaherty BP. Understanding ageing: Further commentary on the limitations of cross-sectional designs for ageing research. Gerontology. 2002;48(1):22–9. 10.1159/000048920.

