# Supplementary material for: Promoting independence in Lewy body dementia through exercise: the PRIDE study
Source: BMC Geriatr. 2022 Aug 9;22:650. doi: 10.1186/s12877-022-03347-2 (PMC9361699; doi:10.1186/s12877-022-03347-2)
Supplement: Supplementary file 1 — Additional file 1: Supplementary Text (S1). detailing Additional Methodology & Results information; Supplementary Tables (S1-S10). detailing the Interpretation of Accelerometry-derived Physical Activity, Gait; Baseline Characteristics of Frailty and Sarcopenia; Accelerometry-derived Gait and Physical Activity Variables; Accelerometry-derived Measures of Gait Quality; Physical Activity and Cognition Associations with Clinical Characteristics at Baseline; Change in Clinical Characteristics during Wait-list period; Change in Clinical Characteristics during Intervention period; Axivity-derived Gait Quality and Quantity Change during Wait-list and Intervention Period; Wait-list Period Non-significant Changes in Accelerometry-derived Gait Quality; and Intervention Period Non-Significant Changes in Accelerometry-derived Gait Quality. This is followed by figures including (in order): and Supplementary Figures (S1 & S2). detailing Consort Flow Diagram; and Average 24-hour activity breakdown – Baseline. [file 12877_2022_3347_MOESM1_ESM.docx]

# Supplementary Material 1

[Supplementary Material 1 1](#_Toc94347419)

[**Supplementary Text S1 - Additional Methodology & Results information** 2](#_Toc94347420)

[Exercise Intervention 2](#_Toc94347421)

[Adverse Events 3](#_Toc94347422)

[Secondary Outcomes 3](#_Toc94347423)

[Axivity Gait Quality and Quantity variables description 4](#_Toc94347424)

[Statistical Analysis 13](#_Toc94347425)

[Results 14](#_Toc94347426)

[Recruitment & Retention 15](#_Toc94347427)

[Adverse Events 15](#_Toc94347428)

[Baseline Characteristics 16](#_Toc94347429)

[Baseline Characteristics 16](#_Toc94347430)

[Intervention Period 21](#_Toc94347431)

[Training Adherence 21](#_Toc94347432)

[**Supplementary Table S1 -** Interpretation of Accelerometry-derived 22](#_Toc94347433)

[Physical Activity, Gait 22](#_Toc94347434)

[**Supplementary Table S2 -** Baseline Characteristics of Frailty and Sarcopenia 24](#_Toc94347435)

[**Supplementary Table S3 -** Accelerometry-derived Gait and Physical Activity Variables 25](#_Toc94347436)

[**Supplementary Table S4 -** Accelerometry-derived Measures of Gait Quality 26](#_Toc94347437)

[**Supplementary Table S5 -** Physical Activity and Cognition Associations with Clinical Characteristics at Baseline 27](#_Toc94347438)

[**Supplementary Table S6 -** Change in Clinical Characteristics during Wait-list period 29](#_Toc94347439)

[**Supplementary Table S7 -** Change in Clinical Characteristics during Intervention period 30](#_Toc94347440)

[**Supplementary Table S8 -** Axivity-derived Gait Quality and Quantity Change during Wait-list and Intervention Period 31](#_Toc94347441)

[**Supplementary Table S9 -** Wait-list Period Non-significant Changes in Accelerometry-derived Gait Quality 33](#_Toc94347442)

[**Supplementary Table S10 -** Intervention Period Non-significant Changes in Accelerometry-derived Gait Quality 36](#_Toc94347443)

[**Supplementary Figure S1** -Consort Flow Diagram 39](#_Toc94347444)

[**Supplementary Figure S2** - Average 24-hour activity breakdown – Baseline 40](#_Toc94347445)

[References 41](#_Toc94347446)

# **Supplementary Text S1 - Additional Methodology & Results information**

### Exercise Intervention

The *Static balance* component was performed for a maximum of 10 minutes of the total session duration inside a custom-built apparatus The participant stood in a position that challenged their postural stability, which was determined by choosing the most challenging balance position (e.g., narrow stance, tandem, etc.) and interacted with the apparatus to encourage and reaching outside of the centre of gravity while performing a variety of progressively more challenging dual-cognitive tasks requiring manual dexterity, visuospatial and executive function.

The *Dynamic Balance* component was performed by instructing the participant to walk in tandem, or as close to that as possible, while performing a combination of physical dual-task (plate holding) and progressively more difficult cognitive tasks, for 5 m along a red tape line with a turn to come back to the start. The assessor closely followed the participant for safety, and provided performance prompts and imagery while monitoring time taken and errors made.

The *Functional Training* component was performed for 5 minutes each session to improve specific functional deficits identified during baseline (i.e. chair stands, transfers) by providing correct technique and cues.

The *Progressive Resistance Training* component involved up to 40 minutes of high intensity machine-based exercises performed with K400 Keiser pneumatic machines (Keiser Sports Health Equipment, Ltd, Fresno, CA, USA). Exercises were performed at an initial intensity of 70% of 1RM, progressing to 80% 1RM in the second session and targeted muscle groups associated with maintaining independence, reducing falls risk, and aiding in posture in older adults. Participants performed 2 sets of 6 repetitions with a focus on a fast concentric and slow eccentric phase at a rating of perceived exercise at or above 8/10. Movements were cued with auditory, visual and tactile stimuli and participants were encourage to focus on the muscle contract where possible to maximise exercise engagement.

### Adverse Events

Monitoring of adverse events and all changes in health status and medical care/interventions was carried out via weekly telephone questionnaires with the caregiver and interview within sessions. Additional information was gathered from medical and nurse care teams if appropriate for participants residing within aged care facilities. Adverse events were defined *a priori* and included any exacerbation of underlying disease, or new onset musculoskeletal, cardiovascular, or metabolic abnormalities. The study geriatrician and ethics committee evaluated all adverse events to adjudicate all events as potentially/definitely related to the study exercise or assessment protocols or not, or any need to change the study protocol.

### Secondary Outcomes

A range of secondary outcomes including *cognition, psychosocial function, quality of life, cardiovascular status, body composition, health status*, *medication interactions*, *physical performance,* *physical activity and sedentary time, exercise capacity and additional functional independence* measures were assessed along with specific *caregiver outcomes including burden and psychosocial state*.

Additionally, a range of variables were derived from raw data. These included chronic conditions, quantity and class of prescribed medications, potentially inappropriate medications (PIMs) as defined by STOPP criteria ^1^, equivalent levodopa dose (calculated with process described in ^2^), anticholinergic cognitive burden scale (ACB) ^3^, Self and proxy reports of weight loss, fatigue, physical activity, physical function scores, strength, and estimated muscle mass and sarcopenia and frailty status of participants (using bioelectrical impedance analysis (BIA) and the equation described in ^4^).

Lastly, accelerometry-derived variables relating to physical activity, gait quantity and quality were collected from a small, accelerometer taped at approximately the level of the L5 vertebral body (Axivity AX3, York, UK; dimensions 23.0 x 32.5 x 7.6 mm; weight: 11g; accuracy 20 parts per million) and initially analysed by co-investigators specialising in biomechanics and accelerometry to derive a range of gait quantity, quality and physical activity variables (*Hausdorff, J, Hillel, I., Movement Disorders Unit, Tel-Aviv Sourasky Medical Center; Department of Physical Therapy, Sackler Faculty of Medicine, Tel-Aviv University, Tel-Aviv, Israel*). Please see **Supplement Table 1** for a thorough description of the collection and analysis of accelerometry variables and interpretation and calculation of each variable.

### Axivity Gait Quality and Quantity variables description

A small, rectangular tri-axial accelerometer (Axivity AX3, York, UK; dimensions 23.0 x 32.5 x 7.6 mm; weight: 11g; accuracy 20 parts per million) was used for accelerometry measurement within this cohort. The monitors were programmed from specialist software OMGUI (<https://github.com/digitalinteraction/openmovement/wiki/AX3-GUI>, Open Lab, Newcastle University, UK) with a sampling rate for triaxial accelerometry data of 100Hz and dynamic range of ±8g. Start time for collection was set to the day following the first assessment at 3am, and the monitor was step to stop collecting data 8 days after starting at 3am. After the monitor was programmed, it was placed inside of a small latex sleeve for additional waterproofing and mounted in an upright orientation centrally on the lumbar region (5^th^ Lumbar segment, spinous process).

The monitor was fixed to the participant with hypoallergenic, waterproof dressing (Opsite transparent adhesive, Smith+Nephew, Watford, Hertfordshire, UK) and caregivers were instructed to check the dressing daily to ensure the fixing was intact and monitor was still in place. Caregivers were also given additional Opsite dressing in case the fixing loosened. Participants and caregivers were given a log sheet to fill out for bed, wake, and daytime nap times, however these data were not used in the subsequent analysis beyond problem solving any issues relating to suspected non-wear or corrupt data. Additionally, the assessor instructed participants and caregivers to continue with normal weekly activity with the exception of avoiding full water immersion activities such as swimming or bathing. Upon return of the monitor, data were cleaned and data were downloaded to the Omgui software program in the form of a continuous wave accelerometer data file (.cwa) file. Upon completion of all participant data points the data files and accompanying scanned log sheets were sent to co-investigator team (*Hausdorff, J, Hillel, I., Movement Disorders Unit, Tel-Aviv Sourasky Medical Center; Department of Physical Therapy, Sackler Faculty of Medicine, Tel-Aviv University, Tel-Aviv, Israel*) for processing. Further rationale for the technical details described below can be found in the supplementary material of Galperin et al 2019 ^5^.

The data extracted from the device were analysed to generate two families of metrics: gait quantity and physical activity, and gait quality. The data were first visually inspected for any erroneous data, non-wear time (monitor removed), or inversion of the monitor (replaced upside down). Two algorithms based on monitor position and activity thresholds respectively ^5^ were applied to the data to automatically differentiate bouts of walking, lying, and standing/sitting. Briefly, the first algorithm transforms the accelerometer data from all three axes into horizontal and vertical coordinates, which allows for discrimination of horizontal (lying) and vertical (sitting, standing, walking) activities as well as setting day end (night time bed time) and day start times (morning out of bed times)^6^. Next, bouts of locomotion were defined by passing the signal through a threshold detector algorithm of 0.5 – 3.0 Hz ^5^. Frequencies in this range enabled classification of periods of locomotion, while those falling below this threshold were classified as passive activities (i.e., standing, sitting).

**Supplementary Table S1** describes the metrics derived from accelerometry that are defined below; along with the unit of measurement and interpretation of values with reference literature provided as available.

#### Gait Quality Measurements

For gait quality measurements walking bouts longer than 30 seconds were used to derive variables relating to the five subdomains of pace, rhythm, symmetry, variability, and complexity. The acceleration data output was analysed in either time or frequency domains to calculate the variables below, which were averaged for all the walking bouts within a given day, and presented as the median daily average for the week.

**Pace** measures included *step length* and its derivative *gait speed*. *Step length* was calculated using the dominant frequency of the peak amplitude within the vertical axis along with participant height to calculate length via the inverted pendulum model ^6^, while *Gait speed* was calculated using step length / step time (described below).

**Rhythm** measures captured the frequency and timing of gait and included *mean step time, step time variability, mean stride time, stride time variability, cadence,* and *dominant frequency of the power spectrum. The dominant frequency of the power spectrum* is calculated in the vertical axis, and represents the dominant frequency of peak amplitudes (i.e., maxima and minima) during gait cycle in the 0.5 – 3.0 Hz locomotion band. *Cadence* (steps/min) was then derived by multiplying the peak frequency (i.e., step peak) by 60 to derive steps/min. The cadence value was then inverted to calculate *step time* (as 60 seconds divided by the step rate yields the time spent in each step) and doubled to calculate *stride times* (as a stride consists of two steps in a cycle). The coefficient of variation (CV) was then calculated by dividing the mean step and stride time for each bout into the standard deviation to derive *step* and *stride time variability*.

**Symmetry** measures captured step-to-step differences between left and right steps within a stride and included *step symmetry, step regularity and harmonic ratio,* which were calculated separately and reported for all three axes (anterior-posterior, medio-lateral and vertical)*. Step symmetry* is a comparative measure of acceleration in the time domain between left and right steps, calculated at an autocorrelation coefficient for each neighbouring contralateral step and averaged across each eligible gait bout. *Step regularity* is also a comparative measure of acceleration in the time domain, but between consecutive steps (i.e., left and left) and represented as an autocorrelation coefficient between each step and the consecutive ipsilateral step averaged across each gait bout. ^7^. The *harmonic ratio* is a measure of overall gait smoothness that is calculated in the frequency domain for each axis. The acceleration profile for each stride is a combination of various regular and irregular acceleration frequencies (harmonics). The dominant frequencies within each stride were identified by decomposition of the signal using Fourier analysis, which breaks down the complex signal into its component frequencies. Regular (or in-phase) acceleration frequencies for the anterior-posterior and vertical axes occur twice or at an even multiple within any given stride cycle (known as even harmonics) due to the biphasic nature of these movements. Irregular (out-of-phase) acceleration frequencies in these axes were therefore identified as frequencies that were not repeated at an even interval within a stride (odd harmonic), and represent for example an extra acceleration signal in one limb but not the other. In the medio-lateral axis, the inverse is true due to the monophasic nature of accelerations within the stride cycle (i.e., accelerations only occur once or at an odd interval multiple). This is due to the alteration between opposite sides in this axis while walking. Therefore, regular (in-phase) frequencies for the ML axis were odd harmonics and irregular (out-of-phase) frequencies were even harmonics. The *harmonic ratio* is as the name suggests, a ratio measure calculated between the sum of regular frequencies compared to the sum of irregular frequencies for at least 20 strides in each axis separately. The higher the sum of regular acceleration frequencies and comparatively lower sum of irregular acceleration frequencies therefore denotes increased smoothness of gait ^8^.

**Variability** measures capture the stride-to-stride differences over a given gait bout and include *stride regularity*, *width of the dominant frequency in the power spectrum, amplitude of the dominant frequency,* and *slope of the dominant frequency. Stride regularity* is a comparative measure of the acceleration in consecutive strides calculated in the time domain with autocorrelation. The *width of the dominant frequency in the power spectrum* is calculated in the frequency domain, and represents the dispersion (width) of the dominant power signal frequency observed within a bout of walking between strides. The *amplitude of the dominant frequency* represents the strength of the dominant signal within a walking bout (i.e., how often the power is generated at a given frequency between strides). The *slope of the dominant frequency* is a combination of the two former measures, whereby the slope of the frequency graph indicates dispersion and periodicity of the power signal during a bout of walking (with a lower slope consisting multiple dominant frequencies having less signal amplitudes indicating greater variability of walking).

**Complexity** measurements capture how variable and regular the gait metrics were with increasing time and include *entropy and sample entropy, and spectral arc length (SPARC). Entropy* quantifies the regularity of gait metrics over time, with more predictable and less complex series having lower entropy values. Likewise, *sample entropy* is a negative logarithmic that quantifies the likelihood that the regularity between two sample gait cycles in the series will be similar to the regularity of another two gait cycles in the series selected by moving along a set distance. In free-living situations, smaller entropy values indicate less change in gait metrics over time and theoretically less responsiveness to environmental stimuli, and are known to be lower in ageing and disease ^9^. *SPARC* provides a measure of movement smoothness over a series in time. The calculation of SPARC can be viewed in more detail in ^9^. Briefly, SPARC is derived from calculating the spectral arc for normalised accelerations in each walking bout and averaging this value across all walking bouts, essentially representing the average variation in acceleration frequencies across the day.

#### Gait Quantity and Physical Activity Measurements

For **gait quantity** measurements, all gait bouts lasting ≥3 seconds were used to quantity *frequency of gait bout durations* (provided for 5-10, 10-20, 20-30, 30-60, 60-120, >120 second bout lengths), *daily step count* and *total daily walking time* (and *proportion of day walking*). These measures were calculated and summed using the frequency domain in the vertical axis to identify acceleration peaks (as described above in the pace and rhythm section).

For **physical activity** measurements, the *signal vector magnitude (SVM)* was used as the primary descriptor of physical activity and represents the measured acceleration signal of all three axes calculated for each 15-second (s) epoch. Specifically, the SVM was derived from the square root of the AP, ML and V axis acceleration squared. The signal was then filtered between 0.5 – 20 Hz using a fourth-order Butterworth band-pass filter and the signal was split into 15-s epochs, whereby the physical activity during the day was the sum of all 15-s epochs throughout the daytime wear hours. Therefore, this measure is a summary measure of total daily physical activity of all epochs throughout the day, and would be higher in more physically active participants. The *proportion of the day active* is calculated by summing the time spent in SVM above a set threshold for activity. This threshold was set as the mean value between the SVM for lying epochs and the SVM for walking epochs. Naturally, the *proportion of the day active* would include all time walking, as well as other unclassified activities, which exceeded the mean threshold unique to each participant.

Additionally, time spent lying and in sitting/standing positions were also calculated from positional and threshold data and was used primarily to provide an estimate of in-bed and out-of-bed cut points to determine physical activity throughout the day. For this analysis, we defined this metric as ‘time spent inactive’ and it was defined as the total time spent in ‘lying time’, ‘sitting/standing time’ or ‘other activities’. Lying time was defined time spent in a position where the mean value of the SVM of the vertical axis was close to zero; sitting/standing time was defined as all time spent still that is not classified as lying; and other activities was defined as activities that involved minimal movement (the SVM was below the median value between lying and walking) but could not be classified as walking, lying, or sitting. We chose to describe this metric as inactivity and not sedentary time for several methodological reasons.

First, our ability to extract data on activities that were considered sedentary was limited due to the placement of the monitor. The lumbar positioning of the monitor meant there were several positional analogues that were not able to be reliably distinguished. For example, the upright position of the monitor on the lumbar region of the back during quiet sitting (a sedentary activity) was unable to be distinguished from the upright position during quiet standing (not a sedentary activity). Similarly, quiet lying time during the day (a sedentary activity) was unable to be distinguished from daytime napping (not considered sedentary due to reported health benefits of napping in some cohorts). Our rationale for including these times in the inactivity category was that participants were more likely to be spending majority of time classified in these periods in ‘sedentary’ behaviours (i.e., more likely to be sitting quietly than standing quietly; more likely to be quietly lying then sleeping), and ultimately all of these activities would likely be metabolically/physiological similar. Second, the algorithm used to detect walking used both positional data and SVM data. Activities that were below the median SVM value between walking and lying were considered undefined physical inactivity, as these activities were very light in nature and infrequent. Conversely, activities that were above the median SVM value between walking and lying were considered part of physically active time. The median daily physically inactive value was calculated from all valid days and presented as the median time spent inactive for the cohort.

**Additional notes on analysis**

All measures derived from accelerometry reflect median values of the daily mean for the week when the monitor was worn, as the data were not normally distributed at the individual level.

In summary, the data represents the following:

1. The mean of each metric on any given day was calculated.
2. As the mean of each day distributed across the 7-day monitoring period was non-normally distributed, the median mean daily was analysed.
3. At the cohort level, due to our small sample size and non-normal distribution of participant data, we subsequently took each individual’s median value (reporting the daily mean) for the week and presented it in descriptive and changes scores as a median value for the cohort.

Thus, all accelerometry-derived values presented represent the median cohort value of each participant’s median weekly value for the daily mean of each variable. Where available, reference values were provided from similar cohorts to guide interpretation of outcomes.

### Statistical Analysis

Data analysis was performed using data analysis software (IBM Corp. Released 2017. IBM SPSS Statistics for Windows, Version 26.0. Armonk, NY: IBM Corp.). Statistical significance was defined as α < 0.05 for all baseline correlations and change score analysis. The data were not normally distributed, as decided through visual inspection of boxplots and the Shapiro-Wilk test. Therefore, data are presented as median (range) or frequencies as appropriate for descriptive statistics, and non-parametric statistics used for change scores.

Descriptive statistics were generated for all relevant baseline data using Spearman’s correlation to first explore associations among functional independence and secondary outcomes with an anticipated relationship and sufficient valid data. Strength of the association was interpreted via the commonly-accepted reference values of small ≤ ±0.2-<0.5, moderate =0.5- <0.8, and strong ≥ ±0.8. A line of best fit was not appropriate, as correlation analysis was performed on ranks not the raw data. Where a mean and standard deviation value were provided in the reference cohort, each individual was compared to the mean to generate a z score, which was then presented within the cohort as the median (range) of z-scores compared to a reference population. For each individual, all measures derived from accelerometry reflect median values of the daily mean for the week when the monitor was worn; as the data were not normally distributed at the individual level across the 7 days of wear (see **Supplementary Table S1** for more detail). Additionally, due to the small sample size of the PRIDE trial, group values presented within the results table also represent the median cohort value. Thus, all accelerometry-derived values presented represent the median participant value of median weekly values for wear.

A non-parametric equivalent of a matched pairs t-test, the Wilcoxon signed rank test^10^, was used to analyse changes in measures over two sequential time periods: 1) baseline to pre-intervention, and 2) pre-intervention to post-intervention, in order to utilise all available participant data. The standardised test statistic (W) for the Wilcoxon signed rank test (W) was calculated on change scores using the following equation:


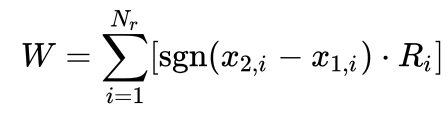


whereby the W statistic is calculated by summing of the difference in positive and negative signs [sign (x_2,I_ – x_1,i_)] multiplied by the ranks (R_i_) of the absolute changes scores. When the W statistic exceeded the critical value, the effect was considered significant^10^. Additionally, confidence interval (CI) estimations on changes scores were calculated using the Hodge-Lehmann’s estimator to generate a median change and confidence interval set at 95% upper and lower bounds.

# Results

## Recruitment & Retention

A total of six participants completed the exercise intervention period out of the nine participants who were enrolled in the waist-list period (**Supplementary Figure S1**). The exercise intervention was undertaken during a period from 20th February 2017 to 8^th^ May 2018.

An additional participant completed the intervention period after a 9-month delay due to multiple clinical events unrelated to the study. His intervention results are reported separately in a published case report^11^.

Two participants dropped out of the study due to ill health. The first participant dropped out following pre-intervention assessment at the conclusion of the wait-list period due to a rapid deterioration in health, which made travel to the exercise facility unfeasible. The second participant undertook 6 weeks of the exercise intervention but had to drop out due to an adverse event unrelated to the study, described in the *adverse events* section below (3.2.2). These two participants compared to the overall sample had greater disease severity and severe cognitive impairment, lower physical function, strength, muscle mass and physical activity, and were at higher risk of malnutrition.

## Adverse Events

Three adverse events occurring in three separate participants were reported during the wait-list control period and were deemed unrelated to the study by the study geriatrician and Ethics Committee (HREC 2). One event was a fractured fibula secondary to a fall in the presence of delirium and occurred in week 8 of the wait-list period. The second event was epistaxis from a fall and suspected head trauma, which also occurred in week 8 of the intervention period. The third was an episode of delirium secondary to faecal impaction, which occurred in week 5 of the wait-list period. None of the events reported occurred during assessment procedures within the study.

A total of two adverse events were recorded during the intervention period, of which only one was potentially related to the study. One adverse event was delirium secondary to faecal impaction and was unrelated to the study but led to the participant dropping out of the intervention. The adverse event deemed potentially related to the study was a minor exacerbation of a pre-existing, small inguinal hernia, which resolved during the study period and only required minor, temporary modification of the intervention (reduction of resistance training load and some lower body exercises). The participant had his hernia assessed by a surgeon upon completing the study, and subsequently the hernia was successfully repaired without incident.

## Baseline Characteristics

### Baseline Characteristics

The characteristics of all participants enrolled at baseline are presented as in **Table 1**. The cohort was predominantly male, with all but two participants living with a dementia diagnosis for at least 12 months. The five participants diagnosed with PDD had been living with a PD diagnosis for 4 -17 years prior to a dementia diagnosis. Six participants were prescribed some combination of levodopa, with seven participants overall prescribed dopaminergic medications with a calculated median levodopa equivalent dose (LED) of 450.0mg (range 26.0 – 1297.5 mg). A neurologist was involved in the diagnosis of all but two participants, with the remaining diagnoses involving a geriatrician and a neuropsychiatrist. All participants except two had a tertiary level education attainment (>12 years education) with two participants having attained post-graduate level qualifications. Former occupations and sectors were varied and included a mixture of unskilled, trade, and managerial positions within the agricultural, administrative, finance, education, and automotive sectors. At the time of baseline assessment five participants resided in their own home or apartment, one in an independent unit in a retirement village, and the remaining three participants resided in aged care facilities. A spouse was the primary caregiver for six of the participants, with adult children providing care for the remaining three participants.

Four participants had a comparable level of functional independence (as measured by FIM) to older adults with advanced PD (Hoehn & Yahr stage IV, mean 45.5 ± 13.7, ^12^, while 5 participants were more functionally independent. Participant motor scores (Part III) scores of the MDS-UPDRS contributed the greatest to overall MDS-UPDRS total score in all participants. The median Part I/II score (out of 104) was 36 (range, 16 – 75), and motor complications (Part IV score, out of 24) were absent in five participants. The severity of dementia in this cohort was mild or moderate in all but two participants according to the CDR algorithm score (**Table 1**), with a median CDR sum score (out of 18) of 5 (range, 3 – 18).

Four participants had scores on the MMSE that were above the reported range for dementia of ≤24 (out of 30)^13^ , however all but one participant had cognitive impairment consistent with dementia (a score ≤80 out of 134) in the disease-specific PD-CRS ^14^. Only six participants were administered the GDS-15 as the remaining participants scored below a level of cognition validated for this depressive symptoms measure (≤10 out of 30 in the MMSE)^14^. None of the interviewed participants scored positively for depressive symptoms on the GDS-15 (>5 out of 15), however three of the interviewed participants and one non-interviewed participant were prescribed antidepressant medications for a history of depression at the time of interview.

There were a total of 45 falls reported by caregivers in the 12 months prior to study contact among the nine participants, of whom only three had not fallen. Only five of the total falls resulted in injury warranting medical attention. Sedation due to medication changes was a factor in two falls, and the main mechanism of falling was forward tripping in four of the reported falls. Five participants from the cohort were prescribed medications with a notable sedative effect, while all but one participant were on dopaminergic medications. A total of 19 potentially inappropriate medication (PIM) violations were found within prescriptions for the cohort. The PIMs mainly involved medications for blood pressure and orthostatic hypotension; neuroleptics/sedatives and falls risk; neuroleptics and blood pressure medication; and prolonged use of neuroleptics/sedatives in participants with history of dementia and/or delirium. Participants in this cohort had a high number of comorbidities (**Table 1**), with the majority being cardiovascular and musculoskeletal. All but one participant was being treated for cardiovascular conditions and/or diseases with hypertension and hyperlipidaemia most common. All participants had previous diagnosis or were diagnosed upon assessment with osteoporosis, osteoarthritis or both conditions.

Additionally, four participants met the criteria for sarcopenia, and all but one participant were either pre-frail or frail as outlined in **Supplementary Table S2**. Median strength values for were 1375 N for bilateral leg press (n=7, range 355 – 3100 N), 240 N for bilateral leg extension (n=7, range 35 – 550 N), and 450 N for triceps extension (n=7, range 150 – 850 N). Skeletal muscle mass (SMM) ranged from 17.85 kg – 36.02 kg with a median value of 27.64 kg. Total balance time median value was 60.84 seconds (range 0 – 75.91 seconds). All but one participant in the cohort was walking at a speed below the commonly cited threshold of 1.0 ms^-1^, at which there is an increased risk of hospitalisation, adverse events and reduced functional independence ^15,16^. Median walking speed during the six-minute walk test was 0.99 m/s (0.16 – 1.99 m/s) and median maximal walking speed measured in the clinic was 1.41 m/s (0.75 – 2.03 m/s). Participants (through self- and/or proxy report) were most frequently engaged in low intensity physical activity involving walking and seated group exercise classes.

**Supplementary Table S3** and **S4** describe accelerometry variables derived from the Axivity monitor worn for 7 days. In contrast to clinically measured gait speed, only three participants had a median free-living gait speed below the sarcopenia threshold in **Supplementary Table S2**. Cadence varied considerably outside of the typical physiological value of 100-115 steps/min with one participant below this range, and four above this range. Stride time variability was significantly higher (worse) than reported values in PD cohorts, with seven participants having a median variability exceeding the mean value reported for individuals with PD characterised as ‘fallers’ [5%, ^17^]. Symmetry and regularity of participant gait between left and right steps (interpreted via the harmonic ratio) varied considerably within the cohort. Nine and six participants from the cohort were more asymmetrical (worse) in the vertical and anterior-posterior axis, respectively, compared to idiopathic PD cohorts ^9^, while only four participants were more asymmetrical (worse) in medio-lateral axis.

Physical activity and gait quantity measures are described together in **Supplementary Table S3** as a summation of overall physical activity in this cohort. Daily physical activity varied greatly and was highly correlated with other proxies for physical activity such as the gait quantity metrics of time spent walking (rho 0.983, P<0.01), step count (rho 0.983, P<0.01), and total bouts of walking (rho 0.983, P <0.01). Median step count of the cohort was 41% of the minimum daily recommendation of 7,000 steps/day for health older adults ^18^, with all but two participants not able to reach this target consistently. Additionally, participants were lying during the day for a median time of 41.4 minutes, (range, 15 - 414.6 minutes). **Supplementary Figure 2** represents that average breakdown of time spent in different activities for the cohort. Participants on average spent 2 hours, 9 minutes (range 2 hrs. 0 min - 7 hrs. 44 min) of the day active while spending 11 hours, 23 minutes (range 9 hrs. 12 min – 13 hrs. 44 min) of the day physically inactive.

Higher levels of physical activity and walking time were significantly associated with better cognition, quality of life, and functional independence (FIM), as well as greater grip and maximal leg strength, walking endurance, habitual and maximal walking speed, muscle mass ratio, and physical function. Better cognition was significantly associated with greater quality of life, functional independence (FIM), walking endurance, physical function, and habitual walking speed. Physical activity, walking time and cognition were inversely associated with dementia severity. Associations are reported in **Supplement S5**.

## Intervention Period

### Training Adherence

The six participants who completed the intervention completed a median of 23 sessions (*range* 19 – 24) out of the maximum 24 sessions allocated, equating to a total of 136 hours of intervention, or an average of 22.6 hours/participant. Participants all completed at least 80% (19/24) of the prescribed sessions. Out of the 6 participants who completed the intervention, 2 independently drove their own car to sessions, 3 participants were transported by their caregivers, and 1 was either driven by his caregiver or caught public transport. Sessions were kept to a duration of one hour as was originally planned, and the order of completing exercises, namely static balance, dynamic balance, PRT, then functional tasks was able to be maintained. Participants did not have significant OFF periods during training, which took place in the morning (AM) as most experienced these periods during the evening, or just upon awakening in the morning.

| **Supplementary Table S1 -** Interpretation of Accelerometry-derivedPhysical Activity, Gait | | | | |
| --- | --- | --- | --- | --- |
| **Domain/family**  Variable | | Axes | Unit | Interpretation |
| **Physical Activity Measures*** | |  | | |
| *Walking time & proportion* | | V | mins, % | Higher duration/proportion is better. McArdle 2019 reports mean walking time in in healthy older adults of 196 ± 63 minutes, and 147 ± 72 and 106 ± 45 minutes in older adults with DLB and PDD. Proportion of the day spent walking was 13 ± 4 %, 10 ± 5 %, and 7 ± 3 % in this population, respectively.^19^ |
| *Total daily physical activity (Sum Vector magnitude)* | | All | g / hr. | Higher sum is better, indicating more accumulated physical activity through the day. |
| *Proportion of day active* | | All | % | Higher proportion is better, however interpretation of the intensity during this ‘activity’s is not possible. |
| *Lying time* | | All | mins | Lying while sleeping during the day is considered beneficial to health, however lying while awake is considered sedentary activity and is better if reduced during daytime. |
| *Proportion of day sitting/standing* | | All | % | Generally, lower is considered better as it is more likely that stationary, upright positional activity in this cohort would unlikely be standing, but more so sitting, which is sedentary. |
| **Gait Quantity**** | |  |  |  |
| *Step count* | | V | n | Higher step count is better. McArdle 2019 reports mean step count of 14204 ± 4817, 10196 ± 5229, and 7305 ± 3018 in healthy older adults, mild DLB, and mild PDD participants respectively ^19^ |
| *Bouts of walking (5-10, 10-20, 20-30, 30-60, 60-120, >120 seconds) & sum of bouts* | | V | n | More walking bouts were better, especially of longer duration. McArdle 2019 reported mean total walking bouts of 630 ± 166, 565 ± 213, and 459 ± 159 in healthy older adults, mild DLB, and mild PDD participants respectively.^19^ |
| **Gait Quality***** | |  | | |
| **Pace** | |  | | |
| *Step length* | | V | m | Longer step length is generally better for a given cadence. McArdle 2019, reports a step length of 0.7 ± 0.09 in healthy older adults and 0.55 ± 0.12 metres in older adults with LBD. ^19^ |
| *Gait speed* | | V | m/s | Faster gait speed is better. In addition to the sarcopenia cut off of 0.8 m/s, McArdle 2019 reports a mean gait speed of 1.26 m/s in healthy, older adults and 0.95 ± 0.24 m/s in older adults with LBD. ^19^ |
| **Rhythm** | |  |  |  |
| *Mean step time & mean stride time,* and | | V | s | Dependent on context. If cadence is the normal physiological range, then step length must also be factored into the interpretation. In PD cohorts, people classified as ‘fallers’ compared to ‘non-fallers’ have been reported to have both increased stride times 1.19 vs. 1.12 seconds ^20^ and reduced stride times (0.92 vs. 1.04 seconds. ^21^ |
| *Step time & stride time variability (CV)* | | V | % | Decreased variability is better. Stride time variability was reported to be 5.0 ± 1.9% in ‘fallers’ in a PD cohort vs. 3.3 ± 1.6% for non-fallers. ^21^ |
| *Cadence* | | V | Steps/min | Normal physiological cadence is 100 – 115 steps/minute ^22^. Older adults and individuals with PD may have to take more, shorter steps to maintain walking speed. |
| *Dominant frequency of the power spectrum.* | | V | Hz | The dominant frequency in the 0.5 – 3.0 Hz locomotion band represents the cadence (steps/min). A dominant frequency approximately in the 1.66 – 1.92 Hz would match the normal physiological cadence. |
| **Symmetry** | |  | | |
| *Step symmetry* | | All | nu | Higher step symmetry (i.e. closer to a value of 1.0) represents more symmetrical, normal steps within the stride. In the most commonly extracted V and ML axis, step symmetry was 0.98 and -0.65 for normal gait, and 0.31 and -0.55 for abnormal gait respectively. ^6^ |
| *Step regularity* | | All | nu | Higher step regularity (i.e. closer to a value of 1.0) represents more regular, normal steps within the stride. In the most commonly extracted V and ML axis, step regularity was 0.89 and -0.85 for normal gait, and 0.26 and -0.55 for abnormal gait respectively. ^6^ |
| *Harmonic ratio* | | All | nu | A higher harmonic ratio indicates a greater proportion of regular to irregular gait acceleration frequencies within the strides cycles, there higher is better. Harmonic ratio in healthy controls vs. a PD cohort was: Vertical – 3.10 ± 0.9 vs. 2.75 ± 0.82, Anterior-Posterior – 2.08 ±0.48 vs. 2.04 ± 0.74, Medio-Lateral – 0.6 ± 0.11 vs. 0.56 ± 0.09 ^9^ |
| **Variability** | |  | | |
| *Stride regularity* | | All | nu | Higher stride regularity (i.e. closer to a value of 1.0) represents more regular, normal strides within the gait. In the most commonly extracted V and ML axis, stride regularity was 0.91 and -0.85 for normal gait, and 0.86 and -0.84 for abnormal gait respectively. ^6^ |
| *Width of the dominant frequency in the power spectrum,* | | All | Hz | A narrower width is better, as it indicates less variation in acceleration produced with each stride – i.e. consistent acceleration signals for each stride within a bout of walking. In a PD cohort classified as ‘fallers’, the frequency width for V, AP and ML axis were 0.7 ± 0.04, 0.7 ± 0.04, and 0.87 ± 0.1 Hz respectively, which was not significantly different from non-fallers ^20^ |
| *Amplitude of the dominant frequency* | | All | g^2^/Hz | Higher amplitudes were indicative or a stronger signal at the dominant frequency of acceleration i.e. – the person spends more time at the dominant frequency during a bout of walking and varies less. Amplitudes in PD participants of 0.58 ± 0.22 (V), 0.68 ± 0.16 (AP) and 0.34 ± 0.25 (ML) in ‘fallers’, and 0.71 ± 0.18, 0.68 ± 0.11, and 0.21 ± 0..13 in ‘non-fallers’ ^20^ |
| *Slope of the dominant frequency* | | All | g^2^/Hz^2^ | A steeper slope/gradient indicates less variability in gait, which is better. A shallow gradient is indicated of smaller amplitudes spread over a wider spectrum of dominant frequencies indicating more variability. The slope in PD participants was reported as 0.20 ± 0.08 (V), 0.22 ± 0.06 (AP) and 0.11 ± 0.09 (ML) in ‘fallers’, and 0.24 ± 0.06, 0.23 ± 0.04, and 0.06 ± 0.04 in ‘non-fallers’ ^20^ |
| **Complexity** | |  | | |
| *Entropy & Sample entropy* | | All | nu | Higher levels of entropy in walking bouts were considered better. Ageing and impaired systems exhibit lower entropy than healthy systems ^23^ |
| *SPARC* | | All | nu | A higher SPARC value indicates an increased smoothness of movement and less variability of acceleration frequencies. ^9^. |
| **Mins**  = minutes, **Hrs.** = Hours, **g** = force of gravity (9.8m^2^), **n** = number, **m** = metres, **m/s**  = metres per second, **Hz** = hertz, **nu** = no unit/unit less  *Physical activity variables were calculated per 15 second epochs, and averaged across all epochs for the day period, **Gait quantity variables were calculated for each bouts of walking and summed across the day, ***Gait quality variables were calculated for each eligible walking bout (>30 seconds) and then average across all walking bouts for a given day. **All variables were reported as the median daily value for the week**. | | | | |

# **Supplementary Table S2 -** Baseline Characteristics of Frailty and Sarcopenia

| Measure | Median (range) | EWGSOP2 Sarcopenia criteria^1^  Threshold | Fried frailty phenotype^2^  threshold |
| --- | --- | --- | --- |
| Highest grip strength – dominant hand, *kg*  *Participants below threshold, n*  Five times sit-to-stand time, *seconds*  *Participants below threshold, n* | 26 (10 - 41)  13.8 (8.13 – 33.72) | *Male <27 kg, Female <16 kg*  4  *Time >15s for five chair rises*  5 | Male <29-32, female <17-21 depending on BMI  5  -  - |
| BIA skeletal muscle Index (SMI), *kgm^-2^*  *Participants below threshold, n* | 9.2 (7.01 – 11.16) | *Male <9.5 kgm^-2^, Female <7.0 kgm^-2^*  4 | -  - |
| Short Physical Performance Battery, */12*  *Participants below threshold, n*  Habitual gait speed, *ms^-1^*  Participants below threshold, *n* | 7 (1 – 12)  0.69 (0.11 – 1.01) | *SPPB total score ≤8*  5  *Gait speed < 0.8 ms^-1^*  5 | -  -  *M ≤173, F ≤159cm, 0.65 ms^-^1, M >173, F >159cm, 0.76 ms^-1^*  5 |
| Reported weight loss in prior year, *%*  Participants exceeding threshold, *n* | 6 (0 – 10.5) | -  - | *Threshold: 5% or 4.5 kg of weight loss in prior year*  5 |
| MDS-UPDRS part I/II, question 13* - fatigue  Participants with positive response, n | 1 (0 – 4) | -  - | *Self-report presence of fatigue ≥1/4^3^*  6 |
| Participants reportedly accruing <150mins of Moderate-Vigorous physical activity per week, *n* | - | -  - | *Low reported weekly physical activity levels*  6 |
| Distance walked in six minutes, *metres* | 358.1 (57 – 718) | - | - |
| MNA-SF score, */14*  Malnourished (0-7), n  At-risk (8-11), *n*  Normal (12-14), *n* | 10 (8 – 13)  0  6  3 | -  -  -  -  - | -  -  -  -  - |
| **Total participants with sarcopenia*, n**  **Low muscle strength and quantity, with or without low physical function*  **Subset satisfying severe sarcopenia criteria*, n**  **Low muscle strength, quantity + physical function* | | **4**  **3** | -  - |
| **Participants classified as Pre-frail (1-2 frailty criteria), n**  **Participants classified as Frail (≥ 3 frailty criteria), n** | | -  - | **2**  **6** |
| All values a presented as **median (range)** or as n participants satisfying criteria **.^1^EWGSOP2** = European Working Group on Sarcopenia in Older People 2 – most recent guidelines on diagnosis and treatment of Sarcopenia, see ^24^. **^2^Fried frailty phenotype** is a widely accepted criteria for frailty involving the domains of weight loss, exhaustion, low physical activity, weakness, and slowness ^25^. **BIA** = Bioelectrical impedance assessment, **MNA-SF** = Mini-Nutritional Assessment – Short Form. **MDS-UPDRS** = Movement Disorder Society Unified Parkinson’s Disease Rating Scale. Part I/II is participants & caregiver rated non-motor and motor experiences of daily living. ^3^Q13 -  *“Over the past week, have you usually felt fatigued? This feeling is not part of being sleepy or sad”* | | | |

# **Supplementary Table S3 -** Accelerometry-derived Gait and Physical Activity Variables

| **Gait Quantity and Physical activity** | **Daily mean value**  Median |
| --- | --- |
| Total daily activity level (g / hour)  *Proportion of day active, %*  Total Walking time, minutes  *Proportion of day walking, %*  Step count, n  Total bouts of waking, n  *Bout lengths*  5 to 10s, n  10 to 20s, n  20 to 30s, n  30 to 60s, n  60 to 120s, n  ≥120s, n | 120.1 (45.4 – 262.7)  10.81 (7.81- 20.29)  46.8 (7.2 – 172.2)  3.25 (0.49- 11.94)  4158 (567 – 12511)  139 (35- 377)  66 (21- 180)  47 (12- 106)  17 (2- 27)  13 (0- 39)  3 (0- 14)  1 (0- 11) |
| All values a presented as **median (range)**. **Proportion** values are calculated a percentage of day spent doing respective activity, whereby the median daily proportion for the week is reported. **N** measures are accumulated daily frequency of respective value, represented as the median daily value for the week. **Total daily activity level** is normalized for each participant to the calculated daytime and represented as an accumulated activity (g, gravity units) per hour, whereby the median daily value over the week is reported. **S** = seconds | |

# **Supplementary Table S4 -** Accelerometry-derived Measures of Gait Quality

| **Gait Quality measures** | **Median of daily mean value**  Median (range) | **Median of daily Standard Deviation values**  Median Mean (range) | |
| --- | --- | --- | --- |
| Pace  *Step length (cm)*  *Gait speed (m/s)*  Rhythm  *Cadence (steps/min)*  *Mean stride time (s)*  *Stride time variability (%)*  *Mean step time (s)*  *Step time variability (%)* | 46.93 (32.43- 68.64)  0.90 (0.62 – 1.29)  114.31 (87.42- 131.9)  1.09 (0.97- 1.41)  8.84 (3.55- 19.74)  0.54 (0.49- 0.7)  12.49 (5.65- 27.2) | 4.66 (3.45- 6.72)  0.11 (0.03- 0.16)  5.36 (3.11- 27.98)  0.06 (0.04- 0.18)  3.04 (2.07- 3.75)  0.03 (0.02- 0.09)  3.75 (2.81- 4.66) |  |
| **Gait Quality measures** | **Anterior-Posterior axis**  Median Mean (range), SD median (range) | **Medio-Lateral axis**  Median Mean (range), SD median (range) | **Vertical axis**  Median mean (range), SD median (range) |
| Symmetry  *Step symmetry (nu)*  *Step regularity (nu)*  *Harmonic ratio (nu)* | 0.17 (0.09- 0.36), 0.13 (0.06- 0.38)  0.48 (0.23- 0.67), 0.08 (0.07- 0.1)  1.62 (1.01- 2.72), 0.24 (0.11- 0.56) | 0.35 (0.26- 0.51), 0.24 (0.16- 0.31)  0.32 (0.21- 0.47), 0.08 (0.07- 0.13)  0.61 (0.49- 0.72), 0.09 (0.07- 0.11) | 0.13 (0.07- 0.37), 0.12 (0.06- 0.16)  0.45 (0.18- 0.73). 0.1 (0.08- 0.15)  1.70 (1.12- 2.67), 0.24 (0.17- 0.57) |
| Variability  *Stride regularity (nu)*  *Width of dominant frequency (Hz)*  *Amplitude of dominant frequency (g^2^/Hz)*  *Slope of dominant frequency (g^2^/Hz^2^)* | 0.47 (0.18- 0.60), 0.08 (0.01- 0.12)  0.73 (0.68- 1.21), 0.19 (0.02- 0.73)  0.59 (0.28- 0.72), 0.09 (0.06- 0.23)  1.11 (0.11- 1.46), 0.22 (0.05- 0.28) | 0.22 (0.16- 0.37), 0.06 (0.05- 0.11)  0.76 (0.69- 0.97), 0.07 (0.02- 0.2)  0.31 (0.14- 0.77), 0.1 (0.06- 0.25)  0.51 (0.18- 1.30), 0.21 (0.1- 0.38) | 0.44 (0.18- 0.68), 0.11 (0.06- 0.14)  0.75 (0.68- 0.83), 0.05 (0.02- 0.15)  0.61 (0.38- 0.92), 0.13 (0.08- 0.26)  1.12 (0.48- 1.91), 0.31 (0.2- 0.39) |
| Complexity  *Entropy (nu)*  *Sample entropy (nu)*  *SPARC (nu)* | 10.08 (8.91- 10.58), 0.26 (0.21- 0.42)  0.19 (0.07- 0.25), 0.04 (0- 0.05)  -32.05 (-50.37- -21.78), 6.08 (1.81- 10.48) | 10.62 (9.22- 10.84), 0.2 (0.15- 0.69)  0.26 (0.09- 0.34), 0.05 (0.01- 0.06)  -44.05 (-64.5- -25.28), 8.99 (5.61- 27.45) | 10.75 (9.35- 11.03), 0.25 (0.03- 0.36)  0.23 (0.08- 0.3), 0.04 (0.01- 0.07)  -29.97 (-54.56- -17.67), 8.55 (5.54- 13.33) |
| All values a presented as **median (range)**. Standard deviation (**SD)** values reported accompany the median day averages, and are reported as a median for the cohort. Accelerometry data were derived from tri-axial accelerometer worn for 8 days (6 valid days). All gait quality measures generated from walking bouts of 30 seconds or longer. **Pace** and **Rhythm** values are first averaged for all eligible walking bouts (>30s) for each day of the week, then the median daily average value is reported for each participant. This Table then reports the median value and range for these values at a cohort level. All three axes are used to quantify movement metrics. **Symmetry, Variability** and **Complexity** measures are calculated with a similar process, except for that the values are reported for each axis separately. **SPARC**  = Spectral Arc Length. **Hz** = Hertz, **Nu** = no unit for measurement, **cm** = centimeters, **g^2/^Hz^2^ =** gravitational units squared per Hertz squared, **s** = seconds. | | | |

# **Supplementary Table S5 -** Physical Activity and Cognition Associations with Clinical Characteristics at Baseline

|  | **Measures of Physical Activity** | | | | **Measures of Cognition** | | | |
| --- | --- | --- | --- | --- | --- | --- | --- | --- |
| *Domain/Measure* | Total walking time | | Total physical activity | | MMSE | | PD-CRS | |
|  | *rho* | *p* | *rho* | *p* | *rho* | *p* | *rho* | *p* |
| Health status | | | | |  | |  | |
| FIM total  MDS-UPDRS total  Part I/II  Part III  CDR sum  MNA-SF | **0.73**  -0.56  **-0.76**  -0.57  **-0.80**  0.44 | **0.026**  0.116  **0.018**  0.112  **0.010**  0.242 | **0.74**  -0.58  **-0.78**  -0.60  **-0.85**  0.36 | **0.021**  0.104  **0.013**  0.088  **0.004**  0.346 | **0.82**  **-0.79**  **-0.84**  **-0.75**  **-0.88**  0.49 | **0.007**  **0.010**  **0.005**  **0.002**  **0.020**  0.183 | **0.80**  **-0.73**  **-0.84**  -0.65  -**0.81**  **0.72** | **0.008**  **0.026**  **0.005**  0.058  **0.08**  **0.028** |
| Psychosocial and quality of life | | | | |  |  |  |  |
| DEMQoL | **0.73** | **0.026** | **0.73** | **0.026** | 0.66 | 0.053 | **0.76** | **0.017** |
| Strength | | | | |  |  |  |  |
| Handgrip Strength  Dominant  Non-dominant  Maximal Leg press | 0.60  **0.82**  **0.86** | 0.088  **0.006**  **0.014** | 0.50  **0.80**  **0.79** | 0.170  **0.010**  **0.036** | 0.12  0.41  0.50 | 0.765  0.271  0.253 | 0.20  0.47  0.57 | 0.606  0.201  0.180 |
| Physical function | | | | |  |  |  |  |
| Gait speed  Habitual  Maximal  Free-living#  Total balance time  SPPB | **0.92**  **0.95**  0.53  **0.84**  **0.94** | **0.001**  **<0.001**  0.139  **0.005**  **0.001** | **0.88**  **0.93**  0.43  **0.82**  **0.94** | **0.002**  **0.001**  0.244  **0.007**  **0.001** | **0.77**  0.60  0.00  **0.69**  **0.77** | **0.016**  0.120  1.00  **0.041**  **0.016** | **0.88**  0.62  0.13  **0.75**  **0.81** | **0.002**  0.102  0.732  **0.019**  **0.008** |
| Exercise capacity | | | | |  |  |  |  |
| 6MWT | **0.92** | **0.001** | **0.90** | **0.001** | **0.68** | **0.042** | **0.77** | **0.016** |
| Body composition | | | | |  |  |  |  |
| SMI | **0.83** | **0.005** | **0.77** | **0.016** | **0.68** | **0.042** | **0.80** | **0.010** |
| Gait quality | | | | |  |  |  |  |
| Symmetry  *Harmonic ratio*  AP  ML  V  Variability  *Entropy*  AP  ML  V  Stride time variability | 0.43  0.12  0.5  0.43  0.33  0.35  -0.23 | 0.244  0.765  0.170  0.244  0.381  0.356  0.546 | 0.32  0.17  0.38  0.33  0.22  0.23  -0.15 | 0.406  0.668  0.308  0.381  0.576  0.546  0.7 | -0.05  0.03  -0.05  0.03  -0.03  -0.12  -0.17 | 0.898  0.932  0.898  0.932  0.932  0.765  0.668 | 0.05  0.13  0.12  0.10  0.22  0.17  -0.10 | 0.898  0.732  0.765  0.798  0.576  0.668  0.798 |
| Footnotes: **Bolded values** indicate significant Spearman’s (rho) Correlation. Significance set at p < 0.05, **FIM**, Functional Independence Measures, **MDS-UPDRS**, Movement Disorder Society Unified Parkinson’s Disease Rating Scale, **CDR**, Clinical Dementia Rating, **MNA-SF**, Mini Nutritional Assessment Short Form, **DEMQoL**, Dementia Quality of Life scale, **SPPB**, Short Physical Performance Battery, **6MWT**, Six-minute walk test, SMI, Skeletal muscle index, **AP**, Antero-posterior axis, **ML**, Medio-lateral axis, **V**, Vertical axis **#** free-living gait speed represents median walking speed while wearing accelerometer in week of monitoring | | | | | | | | |

# **Supplementary Table S6 -** Change in Clinical Characteristics during Wait-list period

| Outcome measure | n | **Baseline**  Median (range) | **Pre - Intervention**  Median (range) | W statistic | Sig. | Median change score | | Hodges-Lehmann CI | |
| --- | --- | --- | --- | --- | --- | --- | --- | --- | --- |
|  | | **8-week wait-list period** | |  | | | | 95% lower | 95% upper |
| CDR sum | 9 | 8 (3 – 18) | 7 (3 – 18) | 1.022 | 0.307 | | 0.5 | -0.5 | 2.25 |
| NPI symptom score  caregiver distress | 9 | 14 (1 – 38)  8 (0 – 18) | 10 (5 – 49)  4 (2 -19) | 0.169  -0.424 | 0.866  0.671 | | 0.5  -0.75 | -8.5  -8 | 15.5  7 |
| Life Space Assessment | 9 | 16 (7 – 34) | 15 (1 – 32) | -1.778 | 0.075 | | -2 | -5 | 0 |
| GDS – 15 score | 6 | 1 (0 – 3) | 2.5 (1 – 6) | 1.633 | 0.102 | | 2 | -1 | 4 |
| DEMQoL – Proxy rating | 9 | 93 (79 – 101) | 90 (71 – 103) | -1.689 | 0.091 | | -2.5 | -11 | 1 |
| SWLS - participant | 6 | 26.5 (12 -33) | 25 (15 – 29) | -0.524 | 0.600 | | -2.5 | -10 | 9.5 |
| MNA-SF total score | 9 | 10 (8 – 13) | 10 (4 – 11) | -1.997 | **0.046** | | -2 | -4.5 | 0.0 |
| Fasting* weight, kg | 9 | 75 (53 – 86.5) | 72 (46 – 84) | -1.901 | 0.057 | | -4 | -6.5 | 0.5 |
| Waist circumference, cm | 9 | 90 (74.5 – 97.5) | 89.8 (70.6 – 98.2) | -1.244 | 0.241 | | -2 | -4.82 | 1.03 |
| Skeletal muscle mass (SMM), kg | 9 | 27.64 (17.85 – 36.02) | 27.48 (16.38 – 35.67) | -1.836 | 0.066 | | -0.51 | -1.31 | 0.30 |
| Skeletal muscle index (SMI), kgm^-2^ | 9 | 9.25 (7.01 – 11.16) | 9.22 (6.73 – 10.65) | -1.955 | 0.051 | | -2.7 | -0.62 | 0.06 |
| Fat mass, kg | 9 | 18.19 (9.23 – 24.35) | 16.49 (10.04 – 18.97) | -1.362 | 0.173 | | -1.65 | -4.90 | 2.54 |
| Fat-free mass, kg | 9 | 54.65 (35.57 – 70.14) | 53.53 (31.98 – 69.19) | -2.547 | **0.011** | | -1.49 | -2.97 | -0.26 |
| Notes: *fasting weight performed in light clothes. Significance values where α < 0.05 are **bolded**. **CDR** = Clinical Dementia Rating, **NPI** = Neuropsychiatric Inventory, **GDS-15** = Geriatric Depression Scale – 15 item, **DEMQoL** = Dementia Quality of Life Scale., **SWLS** = Satisfaction With Life Scale, **MNA-SF** = Mini Nutritional Assessment, **SPPB** = Short Physical Performance Battery, **kg** = kilogram, **kgm^-1^** = kilogram/metre, **s** = second, **cm** = centimeter, **N** = Newton, **N·m** = Newton metre. | | | | | | | | | |

# **Supplementary Table S7 -** Change in Clinical Characteristics during Intervention period

| Outcome measure | n | **Pre - Intervention**  Median (range) | **Post - Intervention**  Median (range) | Wilcoxon Signed Rank standardized test statistic | Sig. | Median change score | | Hodges-Lehmann Confidence interval | |
| --- | --- | --- | --- | --- | --- | --- | --- | --- | --- |
|  | | **8-week Intervention period** | |  | | | | 95% lower | 95% upper |
| CDR sum | 6 | 5 (3 – 18) | 4.75 (1.5 – 18) | -0.921 | 0.357 | | -0.5 | -1.5 | 0.5 |
| NPI symptom score  caregiver distress | - | - | - |  |  | |  |  |  |
| Life Space Assessment | 6 | 20 (10 – 32) | 19.5 (7 – 34) | -0.921 | 0.357 | | -0.5 | -2.5 | 1 |
| GDS – 15 score | 5 | 3 (1 – 6) | 4 (1 – 7) | 0.736 | 0.736 | | 0.5 | -2 | 3 |
| DEMQoL – Proxy rating | 6 | 92 (84 – 103) | 96 (82 – 100) | 0.405 | 0.686 | | 0.5 | -3.5 | 8.5 |
| SWLS - participant | 5 | 25 (15 -29) | 17 (12 – 34) | -0.412 | 0.68 | | -2 | -16 | 5 |
| MNA-SF total score | 6 | 10 (5 – 11) | 11 (9 – 13) | 1.826 | 0.068 | | 1.5 | 0 | 3.5 |
| Fasting* weight, kg | 6 | 73.5 (62 – 84) | 74.5 (70.2 – 87.0) | 1.826 | 0.068 | | 2 | 0 | 5.6 |
| Waist circumference, cm | 5 | 89.47 (83.3 – 98.2) | 90.3 (79.9 – 97.3) | -1.214 | 0.225 | | -1 | -3.4 | 0.4 |
| Skeletal muscle mass (SMM), kg | 5 | 29.06 (23.93 – 35.67) | 30.38 (27.05 – 36.16) | 1.214 | 0.225 | | 0.49 | -0.43 | 1.43 |
| Skeletal muscle index (SMI), kgm^-2^ | 5 | 9.68 (7.39 – 10.65) | 9.92 (9.21 – 11.26) | 1.084 | 0.279 | | 0.15 | -0.15 | 1.22 |
| Fat mass, kg | 5 | 17.27 (11.30 – 24.35) | 16.55 (10.04 – 18.97) | 0.944 | 0.345 | | 0.505 | -0.77 | 1.78 |
| Fat-free mass, kg | 5 | 57.96 (45.39 – 69.19) | 58.78 (53.60 – 70.41) | 1.483 | 0.138 | | 0.77 | -0.47 | 2.52 |
| Notes: *fasting weight performed in light clothes. Significance values where α < 0.05 are **bolded**. **CDR** = Clinical Dementia Rating, **NPI** = Neuropsychiatric Inventory, **GDS-15** = Geriatric Depression Scale – 15 item, **DEMQoL** = Dementia Quality of Life Scale, **SWLS** = Satisfaction With Life Scale, **MNA-SF** = Mini Nutritional Assessment, **SPPB** = Short Physical Performance Battery, **kg** = kilogram, **kgm^-1^** = kilogram/metre, **s** = second, **cm** = centimeter, **N** = Newton, **N·m** = Newton metre. Missing values indicate <5 participant values available and statistical test could not be run. | | | | | | | | | |

# **Supplementary Table S8 -** Axivity-derived Gait Quality and Quantity Change during Wait-list and Intervention Period

|  | |  | |  | |  | | |  | |  |  | |  | |  |  |
| --- | --- | --- | --- | --- | --- | --- | --- | --- | --- | --- | --- | --- | --- | --- | --- | --- | --- |
| **Variable name** | | **Variable code** | | **Median (range)** | | **Median (range)** | | | **Number**  **for analysis** | | **W stat** | **Sig.** | | **Hodge-Lehmann**  **median** | | **95%**  **Lower** | **95%**  **Upper** |
| **Gait Quality metrics** | | | |  | |  | | |  | |  |  | |  | |  |  |
|  | |  | | *Baseline* | | *Pre – Intervention* | | |  | |  |  | |  | |  |  |
| **Wait-list period (n=9)**  *SD od Spectral Arc length V* | | SparcV_STD | | 8.55 (5.54- 13.33) | | 6.2 (0- 9.87) | | | 9 | | -2.073 | 0.038 | | -2.316 | | -7.422 | -0.098 |
| *SD width of dom. freq. V* | | wdV_STD | | 0.05 (0.02- 0.15) | | 0.04 (0- 0.1) | | | 9 | | -2.073 | 0.038 | | -0.035 | | -0.64 | -0.001 |
|  | |  | |  | |  | | |  | |  |  | |  | |  |  |
|  |  | |  | *Pre- Intervention* |  | | *Post- Intervention* |  | |  | | |  | |  | | |
| **Intervention period (n=6)** | | | | | | | | | | | | | | | | | |
| *Amplitude SD* | | ampAP_STD | | 0.08 (0- 0.1) | | 0.12 (0.09- 0.25) | | | 5 | | 2.023 | 0.043 | | 0.093 | | 0.003 | 0.144 |
| *Amplitude SD* | | ampML_STD | | 0.06 (0- 0.14) | | 0.11 (0.07- 0.13) | | | 5 | | 2.023 | 0.043 | | 0.064 | | 0.028 | 0.115 |
| *SD of Entropy in ML axis* | | EntropyML_STD | | 0.18 (0- 0.32) | | 0.26 (0.25- 0.33) | | | 5 | | 2.203 | 0.043 | | 0.171 | | 0.08 | 0.319 |
| *Average step time* | | MeanPeaks | | 0.55 (0.52- 0.59) | | 0.6 (0.57- 0.67) | | | 5 | | 2.023 | 0.043 | | 0.056 | | 0.015 | 0.135 |
| *SD of Slope of dom. Freq. power spectrum AP* | | slpAP_STD | | 0.2 (0- 0.26) | | 0.28 (0.22- 0.62) | | | 5 | | 2.023 | 0.043 | | 0.196 | | 0.014 | 0.365 |
| *SD of Slope of dom. Freq. power spectrum ML* | | slpML_STD | | 0.1 (0- 0.25) | | 0.25 (0.17- 0.25) | | | 5 | | 2.023 | 0.043 | | 0.164 | | 0.078 | 0.251 |
| *Step asymmetry ML* | | stepAsymML | | 0.41 (0.29- 0.56) | | 0.34 (0.26- 0.4) | | | 5 | | -2.023 | 0.043 | | -0.074 | | -0.308 | -0.042 |
| *Mean step time* | | stepTime | | 0.52 (0.35- 0.53) | | 0.58 (0.53- 0.69) | | | 5 | | 2.023 | 0.043 | | 0.11 | | 0.03 | 0.23 |
| *SD of mean step time* | | stepTime_STD | | 0.03 (0- 0.04) | | 0.03 (0.03- 0.23) | | | 5 | | 2.023 | 0.043 | | 0.038 | | 0.001 | 0.202 |
| *SD of Step regularity AP* | | stpRegAP_STD | | 0.05 (0- 0.09) | | 0.09 (0.07- 0.14) | | | 5 | | 2.023 | 0.043 | | 0.049 | | 0.008 | 0.09 |
| *SD of mean stride time* | | strideTime_STD | | 0.05 (0- 0.08) | | 0.06 (0.05- 0.48) | | | 5 | | 2.023 | 0.043 | | 0.075 | | 0.005 | 0.424 |
|  | |  | |  | |  | | |  | |  |  | |  | |  |  |
| Footnote: All variables presented in this table are significant to α < 0.05. Analysis for wait-list period involving all 9 participants, where as analysis for the intervention period involved six participants. A separate set of Wilcoxon-sign (W) test was performed at each time point to maximise participant data. | | | | | | | | | | | | | | | | | |

| **Supplementary Table S9 -** Wait-list Period Non-significant Changes in Accelerometry-derived Gait Quality | | | |
| --- | --- | --- | --- |
| **BL TO PRE (N=9)** | |  |  |
| **Variable name** | | **BL median (range)** | **PRE median (range)** |
| **Gait Quality metrics** |  |  |  |
| *AP amplitude* | | 0.59 (0.28- 0.72) | 0.59 (0.25- 0.7) |
| *Amplitude SD* | | 0.09 (0.06- 0.23) | 0.08 (0- 0.1) |
| *ML amplitude* | | 0.31 (0.14- 0.77) | 0.24 (0.12- 0.5) |
| *Amplitude SD* | | 0.1 (0.06- 0.25) | 0.08 (0- 0.14) |
| *V amplitude* | | 0.61 (0.38- 0.92) | 0.67 (0.2- 0.92) |
| *Amplitude SD* | | 0.13 (0.08- 0.26) | 0.13 (0- 0.17) |
| *Cadence (Frq)* | | 114.31 (87.42- 131.9) | 117.24 (110.38- 140.69) |
| *Cadence SD* | | 5.36 (3.11- 27.98) | 5.02 (0- 8.86) |
| *Cadence (time)* | | 111.5 (87.73- 117.77) | 112.09 (90.1- 117.15) |
| *Cadence SD* | | 5.74 (4.07- 14.24) | 5.56 (0- 8.59) |
| *Variance peak amplitude* | | 0.36 (0.28- 0.42) | 0.32 (0.27- 0.47) |
| *Variance peak amplitude SD* | | 0.07 (0.03- 0.09) | 0.05 (0- 0.08) |
| *Peaks variance* | | 0.3 (0.09- 0.4) | 0.26 (0.11- 0.44) |
| *Peaks variance SD* | | 0.14 (0.07- 0.24) | 0.11 (0- 0.2) |
| *Variance step length* | | 15.77 (6.56- 23.67) | 11.78 (7.9- 26.95) |
| *Variance step length SD* | | 5.25 (0.96- 7.39) | 4.49 (0- 6.85) |
| *Variance step time* | | 12.49 (5.65- 27.2) | 8.47 (5.55- 29.51) |
| *Variance step time SD* | | 3.75 (2.81- 4.66) | 3.28 (0- 5.42) |
| *Variance stride time* | | 8.84 (3.55- 19.74) | 6.03 (4.1- 22.2) |
| *Variance stride time SD* | | 3.04 (2.07- 3.75) | 2.88 (0- 4.35) |
| *Entropy in AP axis* | | 10.08 (8.91- 10.58) | 9.93 (8.25- 10.53) |
| *SD of Entropy in AP axis* | | 0.26 (0.21- 0.42) | 0.23 (0- 0.44) |
| *Entropy in ML axis* | | 10.62 (9.22- 10.84) | 10.6 (9.33- 10.91) |
| *SD of Entropy in ML axis* | | 0.2 (0.15- 0.69) | 0.18 (0- 0.32) |
| *Entropy of V axis* | | 10.75 (9.35- 11.03) | 10.6 (8.76- 11.01) |
| *SD of Entropy in V axis* | | 0.25 (0.03- 0.36) | 0.23 (0- 0.38) |
| *Dominant Frequency in AP axis* | | 1.86 (1.4- 2.08) | 1.89 (1.54- 2) |
| *SD of dominant Frequency in AP axis* | | 0.09 (0.06- 1.14) | 0.09 (0- 0.23) |
| *Dominant Frequency in ML axis* | | 0.92 (0.7- 1.05) | 0.92 (0.85- 1) |
| *SD of dominant Frequency in ML axis* | | 0.05 (0.04- 0.24) | 0.05 (0- 0.09) |
| *Dominant Frequency in V axis* | | 1.91 (1.46- 2.2) | 1.95 (1.84- 2.34) |
| *SD of Dominant Frequency in v axis* | | 0.09 (0.05- 0.47) | 0.08 (0- 0.15) |
| *Harmonic ratio AP* | | 1.62 (1.01- 2.72) | 1.83 (1.13- 2.37) |
| *SD of Harmonic ratio AP* | | 0.24 (0.11- 0.56) | 0.24 (0- 0.56) |
| *Harmonic ratio ML* | | 0.61 (0.49- 0.72) | 0.57 (0.51- 0.7) |
| *SD of Harmonic ratio ML* | | 0.09 (0.07- 0.11) | 0.07 (0- 0.1) |
| *Harmonic ratio V* | | 1.7 (1.12- 2.67) | 1.71 (1.08- 2.51) |
| *SD of Harmonic ratio V* | | 0.24 (0.17- 0.57) | 0.33 (0- 0.52) |
| *Average peaks in amplitude* | | 0.35 (0.14- 0.59) | 0.33 (0.1- 0.66) |
| *SD of average peaks in amplitude* | | 0.08 (0.02- 0.12) | 0.08 (0- 0.11) |
| *Average distance of step* | | 22.84 (15.7- 32.85) | 23.19 (12- 31.46) |
| *SD of average distance of step* | | 3.02 (1.37- 4.34) | 3.1 (0- 4.86) |
| *Average step time* | | 0.56 (0.54- 0.72) | 0.55 (0.52- 0.59) |
| *SD of average step time* | | 0.05 (0.04- 0.11) | 0.05 (0- 0.07) |
| *Average step length* | | 46.93 (32.43- 68.64) | 44.66 (25- 65.76) |
| *SD of average step length* | | 4.66 (3.45- 6.72) | 4.31 (0- 6.92) |
| *Average step times* | | 0.54 (0.49- 0.7) | 0.53 (0.5- 0.55) |
| *SD of average step times* | | 0.03 (0.02- 0.09) | 0.03 (0- 0.04) |
| *Average stride times* | | 1.09 (0.97- 1.41) | 1.07 (1- 1.11) |
| *SD of average stride times* | | 0.06 (0.04- 0.18) | 0.05 (0- 0.08) |
| *Root mean square AP* | | 0.26 (0.12- 0.45) | 0.32 (0.12- 0.6) |
| *SD of Root mean square AP* | | 0.04 (0.02- 0.09) | 0.03 (0- 0.05) |
| *Root mean square ML* | | 0.15 (0.13- 0.21) | 0.17 (0.09- 0.19) |
| *SD of Root mean square ML* | | 0.02 (0.01- 0.03) | 0.02 (0- 0.03) |
| *Root mean square V* | | 0.99 (0.89- 1.02) | 0.98 (0.83- 1.02) |
| *SD of Root mean square V* | | 0.01 (0- 0.02) | 0.01 (0- 0.02) |
| *Range of forces AP* | | 0.95 (0.47- 1.36) | 0.91 (0.45- 1.35) |
| *SD of range of forces AP* | | 0.19 (0- 0.31) | 0.23 (0- 0.29) |
| *Range of forces ML* | | 1.18 (0.59- 1.68) | 1.24 (0.38- 1.68) |
| *SD of range of forces ML* | | 0.25 (0.1- 0.51) | 0.27 (0- 0.47) |
| *Range of forces V* | | 1.26 (0.57- 1.61) | 1.09 (0.35- 1.74) |
| *SD of range of forces V* | | 0.34 (0.11- 0.69) | 0.33 (0- 0.55) |
| *Sample Entropy AP* | | 0.19 (0.07- 0.25) | 0.17 (0.04- 0.25) |
| *SD of Sample Entropy AP* | | 0.04 (0- 0.05) | 0.03 (0- 0.05) |
| *Sample Entropy ML* | | 0.26 (0.09- 0.34) | 0.28 (0.07- 0.36) |
| *SD of Sample Entropy ML* | | 0.05 (0.01- 0.06) | 0.05 (0- 0.06) |
| *Sample Entropy V* | | 0.23 (0.08- 0.3) | 0.24 (0.05- 0.31) |
| *SD of Sample Entropy V* | | 0.04 (0.01- 0.07) | 0.06 (0- 0.07) |
| *Slope of dom. Freq. power spectrum AP* | | 1.11 (0.11- 1.46) | 1.14 (0.34- 1.34) |
| *SD of Slope of dom. Freq. power spectrum AP* | | 0.22 (0.05- 0.28) | 0.21 (0- 0.26) |
| *Slope of dom. Freq. power spectrum ML* | | 0.51 (0.18- 1.3) | 0.46 (0.08- 0.95) |
| *SD of Slope of dom. Freq. power spectrum ML* | | 0.21 (0.1- 0.38) | 0.15 (0- 0.25) |
| *Slope of dom. Freq. power spectrum V* | | 1.12 (0.48- 1.91) | 1.3 (0.22- 1.88) |
| *SD of Slope of dom. Freq. power spectrum V* | | 0.31 (0.2- 0.39) | 0.32 (0- 0.41) |
| *Spectral Arc length AP* | | -32.05 (-50.37- -21.78) | -27.71 (-46.99- -25) |
| *SD od Spectral Arc length AP* | | 6.08 (1.81- 10.48) | 5.93 (0- 8.47) |
| *Spectral Arc length ML* | | -44.05 (-64.5- -25.28) | -43.07 (-73.25- -30.2) |
| *SD od Spectral Arc length ML* | | 8.99 (5.61- 27.45) | 6.05 (0- 10.55) |
| *Spectral Arc length V* | | -29.97 (-54.56- -17.67) | -27.17 (-70.84- -18.87) |
| *SD of peak amplitude* | | 0.14 (0.05- 0.17) | 0.12 (0.03- 0.19) |
| *SD of SD in peak amplitude* | | 0.04 (0.01- 0.11) | 0.04 (0- 0.08) |
| *SD of step time* | | 0.18 (0.05- 0.25) | 0.14 (0.06- 0.26) |
| *SD of SD step time* | | 0.1 (0.06- 0.17) | 0.08 (0- 0.14) |
| *Step asymmetry AP* | | 0.17 (0.09- 0.36) | 0.16 (0.11- 0.32) |
| *SD of step asymmetry AP* | | 0.13 (0.06- 0.38) | 0.1 (0- 0.15) |
| *Step asymmetry ML* | | 0.35 (0.26- 0.51) | 0.42 (0.29- 1.56) |
| *SD of step asymmetry ML* | | 0.24 (0.16- 0.31) | 0.19 (0- 0.32) |
| *Step asymmetry V* | | 0.13 (0.07- 0.37) | 0.11 (0.01- 0.38) |
| *SD of step asymmetry V* | | 0.12 (0.06- 0.16) | 0.1 (0- 0.22) |
| *Mean step time* | | 0.53 (0.47- 0.71) | 0.52 (0.35- 0.54) |
| *SD of mean step time* | | 0.03 (0.02- 0.2) | 0.03 (0- 0.04) |
| *Gait speed* | | 89.8 (62.39- 128.48) | 91.04 (48.31- 124.51) |
| *SD of gait speed* | | 11.34 (3.1- 16.24) | 11.66 (0- 17.85) |
| *Step regularity AP* | | 0.48 (0.23- 0.67) | 0.54 (0.32- 0.65) |
| *SD of Step regularity AP* | | 0.08 (0.07- 0.1) | 0.08 (0- 0.09) |
| *Step regularity ML* | | 0.32 (0.21- 0.47) | 0.31 (0.2- 0.56) |
| *SD of Step regularity ML* | | 0.08 (0.07- 0.13) | 0.07 (0- 0.09) |
| *Step regularity V* | | 0.45 (0.18- 0.73) | 0.5 (0.12- 0.71) |
| *SD of Step regularity V* | | 0.1 (0.08- 0.15) | 0.09 (0- 0.13) |
| *Mean stride time* | | 1.06 (0.95- 1.42) | 1.05 (0.89- 1.12) |
| *SD of mean stride time* | | 0.05 (0.03- 0.28) | 0.05 (0- 0.08) |
| *Stride regularity AP* | | 0.47 (0.18- 0.6) | 0.5 (0.25- 0.6) |
| *SD of Stride regularity AP* | | 0.08 (0.01- 0.12) | 0.09 (0- 0.11) |
| *Stride regularity ML* | | 0.22 (0.16- 0.37) | 0.21 (0.08- 0.43) |
| *SD of Stride regularity ML* | | 0.06 (0.05- 0.11) | 0.06 (0- 0.09) |
| *Stride regularity V* | | 0.44 (0.18- 0.68) | 0.52 (0.14- 0.67) |
| *SD of Stride regularity V* | | 0.11 (0.06- 0.14) | 0.12 (0- 0.14) |
| *Width of dom. freq. AP* | | 0.73 (0.68- 1.21) | 0.72 (0.69- 1.49) |
| *SD width of dom. freq. AP* | | 0.19 (0.02- 0.73) | 0.09 (0- 0.22) |
| *Width of dom. freq. ML* | | 0.76 (0.69- 0.97) | 0.72 (0.7- 0.79) |
| *SD width of dom. freq. ML* | | 0.07 (0.02- 0.2) | 0.04 (0- 0.09) |
| *Width of dom. freq. V* | | 0.75 (0.68- 0.83) | 0.73 (0.69- 1.84) |
| Footnote: All variables presented in this table are not significant to p ≥ 0.05. Analysis for wait-list period involving all 9 participants, where as analysis for the intervention period involved six participants. A separate set of Wilcoxon-sign (W) test was performed at each time point to maximise participant data. | | | |

| **Supplementary Table S10 -** Intervention Period Non-significant Changes in Accelerometry-derived Gait Quality | | | |
| --- | --- | --- | --- |
| **Variable name** | | **PRE median (range)** | **POST median (range)** |
| **Gait quality metrics** |  |  |  |
| *AP amplitude* | | 0.59 (0.25- 0.7) | 0.74 (0.42- 1.03) |
| *ML amplitude* | | 0.32 (0.12- 0.5) | 0.52 (0.25- 0.71) |
| *V amplitude* | | 0.55 (0.2- 0.81) | 0.33 (0.31- 0.41) |
| *Amplitude SD* | | 0.11 (0- 0.17) | 0.08 (0.06- 0.12) |
| *Cadence (Frq)* | | 117.98 (113.55- 140.69) | 108.19 (101.6- 120.18) |
| *Cadence SD* | | 5.18 (0- 8.18) | 6.87 (4.49- 26.35) |
| *Cadence (time)* | | 111.07 (90.1- 113.11) | 103.89 (95.15- 107.87) |
| *Cadence SD* | | 5.47 (0- 8.59) | 4.62 (3.68- 5.06) |
| *Variance peak amplitude* | | 0.33 (0.29- 0.47) | 0.35 (0.28- 0.4) |
| *Variance peak amplitude SD* | | 0.04 (0- 0.08) | 0.05 (0.04- 0.05) |
| *Peaks variance* | | 0.26 (0.11- 0.44) | 0.24 (0.11- 0.43) |
| *Peaks variance SD* | | 0.1 (0- 0.2) | 0.09 (0.08- 0.14) |
| *Variance step length* | | 13.45 (10.49- 26.95) | 12.13 (8.66- 24.43) |
| *Variance step length SD* | | 3.19 (0- 6.43) | 4.46 (2.89- 8.83) |
| *Variance step time* | | 9.4 (7- 29.51) | 8.78 (6.26- 19.85) |
| *Variance step time SD* | | 2.7 (0- 4.09) | 4.11 (2.36- 5.4) |
| *Variance stride time* | | 6.82 (5.29- 22.2) | 7.12 (4.94- 14.58) |
| *Variance stride time SD* | | 2.27 (0- 3.34) | 3.22 (1.62- 4.02) |
| *Entropy in AP axis* | | 9.88 (8.25- 10.43) | 9.72 (8.71- 9.96) |
| *SD of Entropy in AP axis* | | 0.23 (0- 0.44) | 0.33 (0.23- 0.37) |
| *Entropy in ML axis* | | 10.14 (9.33- 10.85) | 9.73 (9.3- 10.14) |
| *Entropy of V axis* | | 10.22 (8.76- 10.96) | 9.6 (9.52- 10.37) |
| *SD of Entropy in V axis* | | 0.2 (0- 0.38) | 0.28 (0.2- 0.34) |
| *Dominant Frequency in AP axis* | | 1.88 (1.54- 1.95) | 1.73 (0.88- 1.9) |
| *SD of dominant Frequency in AP axis* | | 0.09 (0- 0.13) | 0.1 (0.08- 0.16) |
| *Dominant Frequency in ML axis* | | 0.91 (0.85- 0.97) | 0.85 (0.72- 0.95) |
| *SD of dominant Frequency in ML axis* | | 0.05 (0- 0.08) | 0.06 (0.04- 0.08) |
| *Dominant Frequency in V axis* | | 1.97 (1.89- 2.34) | 1.8 (1.69- 2) |
| *SD of Dominant Frequency in v axis* | | 0.09 (0- 0.14) | 0.11 (0.07- 0.44) |
| *Harmonic ratio AP* | | 1.72 (1.13- 2.37) | 1.65 (1.27- 1.94) |
| *SD of Harmonic ratio AP* | | 0.19 (0- 0.56) | 0.26 (0.14- 0.36) |
| *Harmonic ratio ML* | | 0.61 (0.56- 0.7) | 0.63 (0.47- 0.68) |
| *SD of Harmonic ratio ML* | | 0.07 (0- 0.09) | 0.08 (0.07- 0.08) |
| *Harmonic ratio V* | | 1.66 (1.08- 2.27) | 1.68 (1.19- 2.14) |
| *SD of Harmonic ratio V* | | 0.24 (0- 0.42) | 0.29 (0.13- 0.4) |
| *Average peaks in amplitude* | | 0.3 (0.1- 0.6) | 0.21 (0.17- 0.3) |
| *SD of average peaks in amplitude* | | 0.05 (0- 0.1) | 0.03 (0.02- 0.06) |
| *Average distance of step* | | 20.22 (12- 30.09) | 14.67 (13.8- 20.8) |
| *SD of average distance of step* | | 2.37 (0- 4.18) | 1.65 (1.21- 1.98) |
| *SD of average step time* | | 0.04 (0- 0.05) | 0.03 (0.02- 0.06) |
| *Average step length* | | 42.24 (25- 63.18) | 42.57 (33.71- 56.16) |
| *SD of average step length* | | 3.04 (0- 6.01) | 2.76 (2- 12.99) |
| *Average step times* | | 0.53 (0.52- 0.54) | 0.59 (0.53- 0.93) |
| *SD of average step times* | | 0.02 (0- 0.04) | 0.03 (0.02- 0.24) |
| *Average stride times* | | 1.07 (1.04- 1.09) | 1.18 (1.07- 1.85) |
| *SD of average stride times* | | 0.05 (0- 0.08) | 0.06 (0.05- 0.47) |
| *Root mean square AP* | | 0.41 (0.12- 0.6) | 0.36 (0.2- 0.5) |
| *SD of Root mean square AP* | | 0.03 (0- 0.05) | 0.05 (0.04- 0.05) |
| *Root mean square ML* | | 0.17 (0.09- 0.18) | 0.13 (0.1- 0.2) |
| *SD of Root mean square ML* | | 0.01 (0- 0.03) | 0.02 (0.01- 0.04) |
| *Root mean square V* | | 0.95 (0.83- 1.02) | 0.92 (0.9- 0.97) |
| *SD of Root mean square V* | | 0.01 (0- 0.02) | 0.02 (0.01- 0.03) |
| *Range of forces AP* | | 0.76 (0.45- 1.33) | 0.75 (0.52- 0.87) |
| *SD of range of forces AP* | | 0.14 (0- 0.23) | 0.11 (0.06- 0.22) |
| *Range of forces ML* | | 0.96 (0.38- 1.68) | 0.59 (0.52- 0.67) |
| *SD of range of forces ML* | | 0.17 (0- 0.47) | 0.11 (0.07- 0.15) |
| *Range of forces V* | | 0.87 (0.35- 1.67) | 0.69 (0.65- 0.79) |
| *SD of range of forces V* | | 0.18 (0- 0.55) | 0.14 (0.09- 0.14) |
| *Sample Entropy AP* | | 0.15 (0.04- 0.24) | 0.11 (0.08- 0.16) |
| *SD of Sample Entropy AP* | | 0.02 (0- 0.05) | 0.02 (0.01- 0.03) |
| *Sample Entropy ML* | | 0.16 (0.07- 0.36) | 0.11 (0.07- 0.16) |
| *SD of Sample Entropy ML* | | 0.03 (0- 0.06) | 0.02 (0.01- 0.03) |
| *Sample Entropy V* | | 0.15 (0.05- 0.31) | 0.1 (0.09- 0.15) |
| *SD of Sample Entropy V* | | 0.03 (0- 0.07) | 0.02 (0.01- 0.03) |
| *Slope of dom. Freq. power spectrum AP* | | 1.15 (0.34- 1.34) | 1.43 (0.61- 1.74) |
| *Slope of dom. Freq. power spectrum ML* | | 0.56 (0.08- 0.95) | 0.73 (0.48- 1.41) |
| *Slope of dom. Freq. power spectrum V* | | 0.88 (0.22- 1.64) | 0.59 (0.38- 0.77) |
| *SD of Slope of dom. Freq. power spectrum V* | | 0.25 (0- 0.4) | 0.2 (0.15- 0.23) |
| *Spectral Arc length AP* | | -29.09 (-46.99- -25) | -25.68 (-40.57- -15.23) |
| *SD od Spectral Arc length AP* | | 5.34 (0- 7.09) | 5.27 (4.7- 10.7) |
| *Spectral Arc length ML* | | -47.19 (-73.25- -30.2) | -36.65 (-41.15- -26.42) |
| *SD od Spectral Arc length ML* | | 5.32 (0- 9.44) | 6.34 (5.53- 7.11) |
| *Spectral Arc length V* | | -32.67 (-70.84- -21.36) | -44.79 (-57.46- -31.4) |
| *SD od Spectral Arc length V* | | 5.53 (0- 9.87) | 7.51 (4.86- 8.56) |
| *SD of peak amplitude* | | 0.09 (0.03- 0.19) | 0.07 (0.07- 0.09) |
| *SD of SD in peak amplitude* | | 0.03 (0- 0.08) | 0.02 (0.02- 0.03) |
| *SD of step time* | | 0.14 (0.06- 0.26) | 0.14 (0.07- 0.27) |
| *SD of SD step time* | | 0.06 (0- 0.14) | 0.07 (0.05- 0.09) |
| *Step asymmetry AP* | | 0.19 (0.11- 0.32) | 0.17 (0.12- 0.33) |
| *SD of step asymmetry AP* | | 0.07 (0- 0.13) | 0.1 (0.06- 0.22) |
| *SD of step asymmetry ML* | | 0.19 (0- 0.32) | 0.2 (0.1- 0.26) |
| *Step asymmetry V* | | 0.13 (0.01- 0.38) | 0.2 (0.14- 0.36) |
| *SD of step asymmetry V* | | 0.1 (0- 0.22) | 0.19 (0.14- 0.24) |
| *Gait speed* | | 78.97 (48.31- 117.74) | 61.03 (57.85- 81.72) |
| *SD of gait speed* | | 8.75 (0- 15.72) | 5.82 (3.81- 7.1) |
| *Step regularity AP* | | 0.53 (0.32- 0.63) | 0.58 (0.35- 0.62) |
| *Step regularity ML* | | 0.3 (0.2- 0.56) | 0.5 (0.32- 0.52) |
| *SD of Step regularity ML* | | 0.07 (0- 0.08) | 0.08 (0.06- 0.1) |
| *Step regularity V* | | 0.49 (0.12- 0.64) | 0.5 (0.23- 0.62) |
| *SD of Step regularity V* | | 0.08 (0- 0.11) | 0.07 (0.06- 0.11) |
| *Mean stride time* | | 1.04 (0.89- 1.12) | 1.16 (1.04- 1.34) |
| *Stride regularity AP* | | 0.51 (0.25- 0.6) | 0.46 (0.3- 0.64) |
| *SD of Stride regularity AP* | | 0.08 (0- 0.11) | 0.1 (0.07- 0.13) |
| *Stride regularity ML* | | 0.21 (0.08- 0.43) | 0.35 (0.23- 0.41) |
| *SD of Stride regularity ML* | | 0.05 (0- 0.09) | 0.08 (0.06- 0.1) |
| *Stride regularity V* | | 0.48 (0.14- 0.6) | 0.37 (0.17- 0.54) |
| *SD of Stride regularity V* | | 0.11 (0- 0.14) | 0.1 (0.06- 0.12) |
| *Width of dom. freq. AP* | | 0.72 (0.69- 1.49) | 0.76 (0.7- 1.12) |
| *SD width of dom. freq. AP* | | 0.07 (0- 0.2) | 0.12 (0.02- 0.24) |
| *Width of dom. freq. ML* | | 0.73 (0.7- 0.79) | 0.77 (0.69- 0.92) |
| *SD width of dom. freq. ML* | | 0.03 (0- 0.09) | 0.07 (0.02- 0.09) |
| *Width of dom. freq. V* | | 0.74 (0.69- 1.84) | 0.74 (0.71- 1.15) |
| *SD width of dom. freq. V* | | 0.04 (0- 0.05) | 0.16 (0.03- 0.21) |
| Footnote: All variables presented in this table are not significant to p ≥ 0.05. Analysis for wait-list period involving all 9 participants, where as analysis for the intervention period involved six participants. A separate set of Wilcoxon-sign (W) test was performed at each time point to maximise participant data. | | | |

# **Supplementary Figure S1** -Consort Flow Diagram


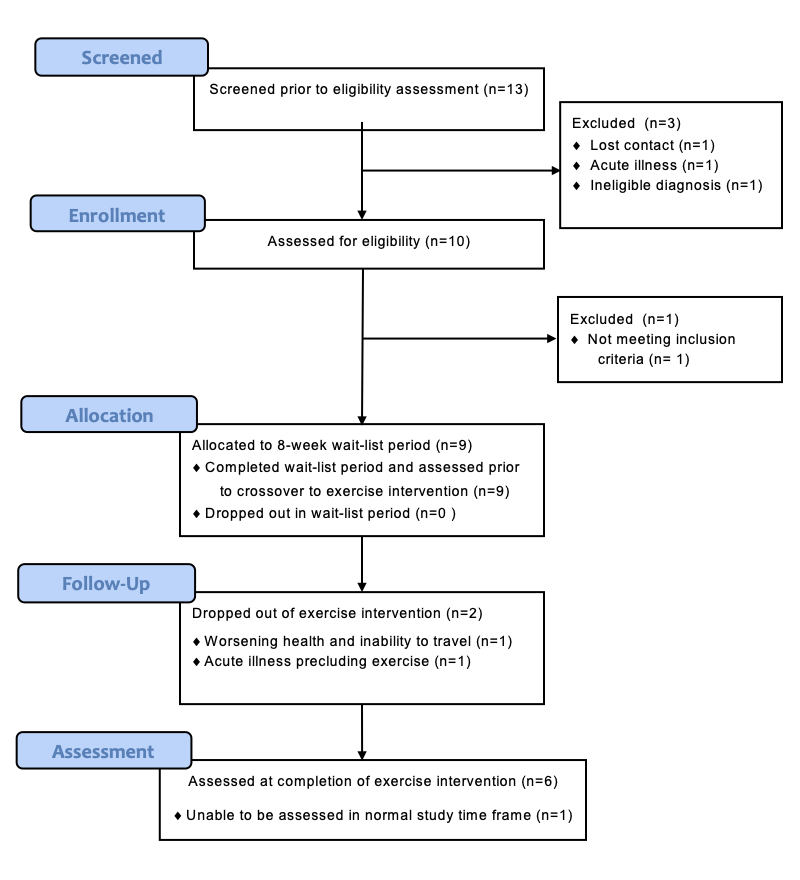


# **Supplementary Figure S2** - Average 24-hour activity breakdown – Baseline

|  |
| --- |
| 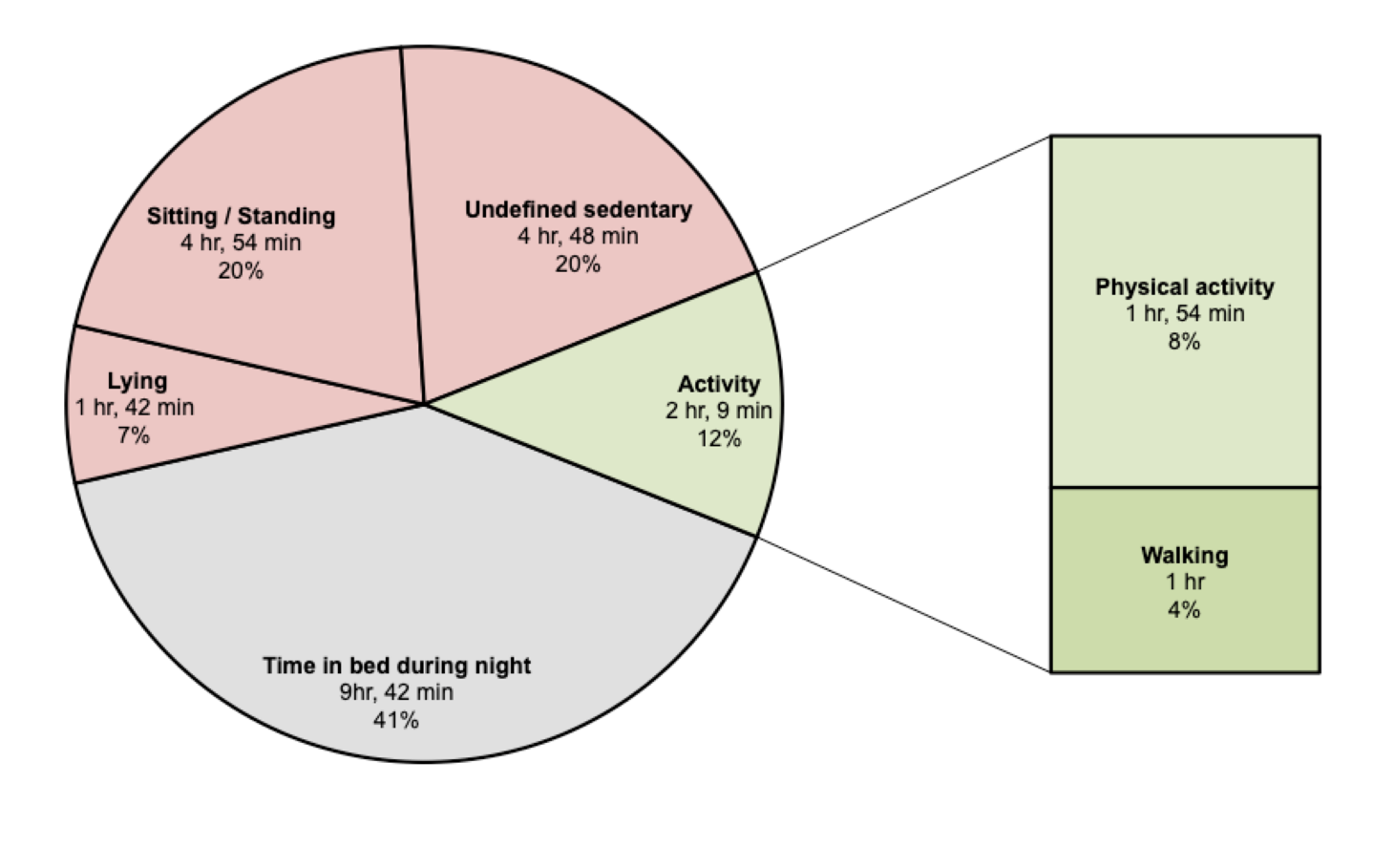 |
| **Note:** Physical activity was defined as time spent at activity levels above activity a threshold set as the mean value between walking and lying activities. Therefore, activities below this threshold including sitting/standing, lying and other undefined activities are defined as physically inactive. Sedentary time could not be precisely defined as standing and daytime napping, which are not considered sedentary behaviour, form part of these categories in red but could not be reliably separated. |

# References

1. Thevelin S, El Mounaouar L, Marien S, Boland B, Henrard S, Dalleur O. Potentially Inappropriate Prescribing and Related Hospital Admissions in Geriatric Patients: A Comparative Analysis between the STOPP and START Criteria Versions 1 and 2. *Drugs & Aging*. 2019:1-7.

2. Tomlinson CL, Stowe R, Patel S, Rick C, Gray R, Clarke CE. Systematic review of levodopa dose equivalency reporting in Parkinson's disease. *Movement disorders*. 2010;25(15):2649-2653.

3. Boustani M, Campbell N, Munger S, Maidment I, Fox C. Impact of anticholinergics on the aging brain: a review and practical application. 2008;

4. Janssen I, Heymsfield SB, Baumgartner RN, Ross R. Estimation of skeletal muscle mass by bioelectrical impedance analysis. *Journal of applied physiology*. 2000;89(2):465-471.

5. Galperin I, Hillel I, Del Din S, et al. Associations between daily-living physical activity and laboratory-based assessments of motor severity in patients with falls and Parkinson's disease. *Parkinsonism & related disorders*. 2019;62:85-90.

6. Moe-Nilssen R, Helbostad JL. Estimation of gait cycle characteristics by trunk accelerometry. *Journal of biomechanics*. 2004;37(1):121-126.

7. Barden JM, Clermont CA, Kobsar D, Beauchet O. Accelerometer-based step regularity is lower in older adults with bilateral knee osteoarthritis. *Frontiers in human neuroscience*. 2016;10:625.

8. Bellanca J, Lowry K, VanSwearingen J, Brach J, Redfern M. Harmonic ratios: a quantification of step to step symmetry. *Journal of biomechanics*. 2013;46(4):828-831.

9. Beck Y, Herman T, Brozgol M, Giladi N, Mirelman A, Hausdorff JM. SPARC: a new approach to quantifying gait smoothness in patients with Parkinson’s disease. *Journal of neuroengineering and rehabilitation*. 2018;15(1):49.

10. Wilcoxon F, Katti S, Wilcox RA. Critical values and probability levels for the Wilcoxon rank sum test and the Wilcoxon signed rank test. *Selected tables in mathematical statistics*. 1970;1:171-259.

11. Inskip M, Mavros Y, Sachdev PS, Singh MAF. Interrupting the trajectory of frailty in dementia with Lewy bodies with anabolic exercise, dietary intervention and deprescribing of hazardous medications. *BMJ Case Reports CP*. 2020;13(4):e231336.

12. Ellis T, Katz DI, White DK, DePiero TJ, Hohler AD, Saint-Hilaire M. Effectiveness of an inpatient multidisciplinary rehabilitation program for people with Parkinson disease. *Physical therapy*. 2008;88(7):812-819.

13. Folstein MF, Folstein SE, McHugh PR. “Mini-mental state”: a practical method for grading the cognitive state of patients for the clinician. *Journal of psychiatric research*. 1975;12(3):189-198.

14. Pagonabarraga J, Kulisevsky J, Llebaria G, García‐Sánchez C, Pascual‐Sedano B, Gironell A. Parkinson's disease‐cognitive rating scale: a new cognitive scale specific for Parkinson's disease. *Movement disorders: official journal of the Movement Disorder Society*. 2008;23(7):998-1005.

15. Kyrdalen IL, Thingstad P, Sandvik L, Ormstad H. Associations between gait speed and well‐known fall risk factors among community‐dwelling older adults. *Physiotherapy research international*. 2019;24(1):e1743.

16. Cesari M, Kritchevsky SB, Penninx BW, et al. Prognostic value of usual gait speed in well‐functioning older people—results from the Health, Aging and Body Composition Study. *Journal of the American Geriatrics Society*. 2005;53(10):1675-1680.

17. Hausdorff JM. Gait dynamics in Parkinson’s disease: common and distinct behavior among stride length, gait variability, and fractal-like scaling. *Chaos: An Interdisciplinary Journal of Nonlinear Science*. 2009;19(2):026113.

18. Tudor-Locke C, Craig CL, Aoyagi Y, et al. How many steps/day are enough? For older adults and special populations. *International Journal of Behavioral Nutrition and Physical Activity*. 2011;8(1):80.

19. Mc Ardle R, Galna B, Donaghy P, Thomas A, Rochester L. Do Alzheimer's and Lewy body disease have discrete pathological signatures of gait? *Alzheimer's & Dementia*. 2019;15(10):1367-1377.

20. Weiss A, Herman T, Plotnik M, Brozgol M, Giladi N, Hausdorff J. An instrumented timed up and go: the added value of an accelerometer for identifying fall risk in idiopathic fallers. *Physiological measurement*. 2011;32(12):2003.

21. Schaafsma JD, Giladi N, Balash Y, Bartels AL, Gurevich T, Hausdorff JM. Gait dynamics in Parkinson's disease: relationship to Parkinsonian features, falls and response to levodopa. *Journal of the neurological sciences*. 2003;212(1-2):47-53.

22. Ogaya S, Higuchi Y, Tanaka M, Fuchioka S. Effect of aging on seated stepping variability. *Journal of physical therapy science*. 2013;25(8):901-903.

23. Costa M, Goldberger AL, Peng C-K. Multiscale entropy analysis of complex physiologic time series. *Physical review letters*. 2002;89(6):068102.

24. Cruz-Jentoft AJ, Baeyens JP, Bauer JM, et al. Sarcopenia: European consensus on definition and diagnosisReport of the European Working Group on Sarcopenia in Older PeopleA. J. Cruz-Gentoft et al. *Age and ageing*. 2010;39(4):412-423.

25. Fried LP, Tangen CM, Walston J, et al. Frailty in older adults: evidence for a phenotype. *The Journals of Gerontology Series A: Biological Sciences and Medical Sciences*. 2001;56(3):M146-M157.
